# Dynamic foot function as a risk factor for lower limb overuse injury: a systematic review

**DOI:** 10.1186/s13047-014-0053-6

**Published:** 2014-12-19

**Authors:** Geoffrey J Dowling, George S Murley, Shannon E Munteanu, Melinda M Franettovich Smith, Bradley S Neal, Ian B Griffiths, Christian J Barton, Natalie J Collins

**Affiliations:** Department of Podiatry, Faculty of Health Sciences, La Trobe University, Melbourne, Australia; Lower Extremity and Gait studies program, Faculty of Health Sciences, La Trobe University, Melbourne, Australia; School of Physiotherapy, Australian Catholic University, Brisbane, Australia; Pure Sports Medicine, London, UK; Centre for Sports and Exercise Medicine, Queen Mary University of London, London, UK; Complete Sports Care, Melbourne, Australia; Department of Mechanical Engineering, Melbourne School of Engineering, The University of Melbourne, Melbourne, Australia

**Keywords:** Biomechanics, Plantar pressures, Kinematics, Prospective studies, Musculoskeletal diseases, Review

## Abstract

**Background:**

Dynamic foot function is considered a risk factor for lower limb overuse injuries including Achilles tendinopathy, shin pain, patellofemoral pain and stress fractures. However, no single source has systematically appraised and summarised the literature to evaluate this proposed relationship. The aim of this systematic review was to investigate dynamic foot function as a risk factor for lower limb overuse injury.

**Methods:**

A systematic search was performed using Medline, CINAHL, Embase and SportDiscus in April 2014 to identify prospective cohort studies that utilised dynamic methods of foot assessment. Included studies underwent methodological quality appraisal by two independent reviewers using an adapted version of the Epidemiological Appraisal Instrument (EAI). Effects were expressed as standardised mean differences (SMD) for continuous scaled data, and risk ratios (RR) for nominal scaled data.

**Results:**

Twelve studies were included (total n = 3,773; EAI 0.44 to 1.20 out of 2.00, representing low to moderate quality). There was limited to very limited evidence for forefoot, midfoot and rearfoot plantar loading variables (SMD 0.47 to 0.85) and rearfoot kinematic variables (RR 2.67 to 3.43) as risk factors for patellofemoral pain; and plantar loading variables (forefoot, midfoot, rearfoot) as risk factors for Achilles tendinopathy (SMD 0.81 to 1.08). While there were significant findings from individual studies for plantar loading variables (SMD 0.3 to 0.84) and rearfoot kinematic variables (SMD 0.29 to 0.62) as risk factors for ‘non-specific lower limb overuse injuries’, these were often conflicting regarding different anatomical regions of the foot. Findings from three studies indicated no evidence that dynamic foot function is a risk factor for iliotibial band syndrome or lower limb stress fractures.

**Conclusion:**

This systematic review identified very limited evidence that dynamic foot function during walking and running is a risk factor for patellofemoral pain, Achilles tendinopathy, and non-specific lower limb overuse injuries. It is unclear whether these risk factors can be identified clinically (without sophisticated equipment), or modified to prevent or manage these injuries. Future prospective cohort studies should address methodological limitations, avoid grouping different lower limb overuse injuries, and explore clinically meaningful representations of dynamic foot function.

**Electronic supplementary material:**

The online version of this article (doi:10.1186/s13047-014-0053-6) contains supplementary material, which is available to authorized users.

## Introduction

Overuse injuries of the lower limb associated with intensive weight bearing exercise are a significant problem for athletes and military recruits, with estimated incidence of running-related injuries reported to range from 20% to 79% [1]. Lower limb overuse injuries are generally recognised as having multifactorial aetiologies [2]. Some of the most common injuries, such as Achilles tendinopathy, medial tibial stress syndrome, patellofemoral pain and lower limb stress fractures, are reported to be more prevalent in those with altered foot function [3,4].

The potential mechanisms linking variations in dynamic foot function with lower limb overuse injury may be related to altered lower limb biomechanics and subsequent changes in tissue stress [5]. This is supported by laboratory-based research using uninjured participants, which suggests that variations in foot posture (flat- and normal-arched feet) are associated with systematic differences in lower limb kinematics [6-8], kinetics [4,9,10], muscle function [11-16] and tendon morphometry [17].

While laboratory-based research is important for understanding potential mechanisms linking foot function and lower limb overuse injury, field-based prospective studies are required to determine whether foot function is a risk factor for lower limb overuse injury. Our accompanying systematic review [18] found that static measures indicating greater foot pronation were associated with an increased risk of patellofemoral pain and medial tibial stress syndrome. However, the small effects suggest that static measures may not adequately represent dynamic foot function. A substantial number of prospective studies have utilised a variety of measurement techniques in order to quantify dynamic foot function and its relationship with lower limb overuse injury [19-46]. However, it is unclear if there are consistent findings across different measures, or whether particular foot function characteristics are risk factors for specific overuse injuries. Enhanced knowledge regarding this may lead to the development of targeted preventative strategies.

Therefore, the aim of this systematic review was to: (i) identify and appraise the current evidence for the prospective link between dynamic foot posture and lower limb overuse injury; and (ii) provide guidance for future research in this area. This review represents the second component of a two-part systematic review on foot posture-related risk factors for lower limb overuse injury.

## Methods

The systematic review protocol was developed in consultation with guidelines provided by the Preferred Reporting of Systematic Reviews and Meta-Analysis (PRISMA) Statement [47].

### Search strategy

MEDLINE, CINAHL, Embase and SPORTDiscus were searched from inception until April 2014. Medical Subject Headings (MeSH) were exploded to include relevant subheadings, in addition to keywords specific to the research question (Additional file 1). The search was limited to adult human participants and English language publications. To ensure identification of all relevant studies, reference lists of appropriate narrative and systematic reviews were hand searched, and discussion with field experts (e.g. physiotherapists, podiatrists) was conducted regarding known important publications. A cited reference search for each included paper was also completed in Google Scholar.

### Eligibility criteria

All studies identified by the search strategy were exported to Endnote version X5 (Thomson Reuters, Philadelphia), by a single investigator (GJD). Abstracts and then full text versions were reviewed by two authors (GJD, MMFS) to determine eligibility. Discrepancies were resolved in consultation with a third reviewer (GSM). Initial eligibility criteria were: (i) prospective cohort study design; (ii) quantitative measurement of foot posture or function at baseline (static or dynamic); and (iii) prospective collection of specific or non-specific lower limb overuse injury surveillance data over a specified time period. Specific lower limb overuse injuries were defined as injuries with a single diagnosis, while non-specific lower limb overuse injuries included injuries without a specific diagnosis or where multiple overuse types of injuries were pooled by the study reviewed. After retrieval of studies that fulfilled the initial eligibility criteria, suitable studies were separated into those that investigated dynamic measures of foot function (i.e. measured during walking or running), and those that investigated static measures of foot posture. This review focused on dynamic measures as risk factors, while static measures are addressed in the accompanying review [18].

### Quality assessment

Assessment of the methodological quality of the included studies was performed using the Epidemiological Appraisal Instrument (EAI) [48]. This instrument is designed to assess the quality of cohort (prospective and retrospective) studies. The EAI consists of 43 items separated into five domains — (i) reporting, (ii) subject/record selection, (iii) measurement quality, (iv) data analysis and (v) generalisability of results [48]. Items on the EAI were scored as “Yes” (score of 2), “Partial” (score of 1), “No” (score of 0), “Unable to determine” (score of 0) or “Not Applicable” (item excluded). The EAI has demonstrated good/excellent validity, and good to excellent intra-rater (Kappa coefficient range 52 to 60), and inter-rater reliability (Kappa coefficient = 90% [95% CI; 87 to 92%]) [48]. For the purpose of this review, the wording of all 43 items was modified slightly to improve clarity and rater interpretation. No items were removed or modified, in order to maintain validity (Additional file 2).

Two raters (GJD, NJC) independently evaluated each study while blind to author and publication details. For any discrepancies in assessment of items between the two raters, a meeting occurred and consensus was achieved. To evaluate the overall quality of the studies, average scores across the 43 items were calculated, with a maximum possible score of two (i.e. as individual items are scored ‘0’, ‘1’ or ‘2’, the maximum ‘average’ score across 43 items is two). A ranking system was used to evaluate the quality of evidence, whereby studies were classified as being high (EAI ≥ 1.4), moderate (EAI 1.1 to <1.4), or low quality (EAI < 1.1) [47].

### Data management

Two investigators (GJD, GSM) extracted data regarding study characteristics, including publication details (year, author, country), participant characteristics (number of injured and uninjured, age, sex, inclusion and exclusion criteria, population [i.e. military]) and study methods (dynamic foot function measurement, examiner details, injury outcome, duration of study and covariates investigated). To facilitate calculation of effects, means and standard deviations (SD) were extracted for injured and uninjured participants for continuous foot function variables, while raw counts were extracted for nominal variables.

Where appropriate data was not provided in the publication, authors were contacted with a request to provide additional data. Where studies described specific variables but did not publish data, it was recorded as ‘not reported’ (NR) and, for the purpose of the analysis, assumed that the variable investigated was not significantly different between the injured and the uninjured population.

### Statistical methods

Inter-rater reliability of the raters’ EAI scores was evaluated using a descriptive analysis. Differences between rater scores for “Yes”, “Partial”, “No”, and “Unable to determine” were calculated, with a difference of zero indicating perfect agreement and a difference of 1 indicating near perfect. The rating “not applicable” was excluded from analysis because no interpretation was required for this rating.

For continuous foot function variables, standardised mean differences (SMD) were calculated as the difference between injured and uninjured group means, divided by the pooled standard deviation [49]. SMDs and 95% confidence intervals (CI) were calculated using the ‘Effect Size Calculator’ from the Centre for Evaluation and Monitoring [50]. Interpretation of the SMD was based on previous recommendations, where > 1.2 was considered large, 0.6 to 1.2 moderate, and < 0.6 small [51]. For nominal scaled foot function variables, risk ratios (RR) and 95% CI were calculated using the ‘Confidence Interval Calculator’ from the Physiotherapy Evidence Database (PEDro) [52]. This was represented as the number of participants with lower limb overuse injury in the group with the associated factor (e.g. delayed time to peak force), divided by participants with lower limb overuse injury in the group without the associated factor. A RR > 1.0 indicated that the lower limb overuse injury was more likely to be found in participants with the risk factor present. A small effect was indicated by a RR ≥ 2.0, and a large effect ≥ 4.0 [53]. Effects were considered statistically significant if the associated 95% CI did not contain zero for the SMD, or one for RR.

### Evidence-based recommendations

In order to provide recommendations based on statistical findings, while incorporating the methodological quality of included papers, a scale regarding levels of evidence was utilised, based on previous work by van Tulder et al. [54].

***Strong evidence:*** pooled results derived from three or more studies, including a minimum of two high quality studies that are statistically homogenous; may be associated with a statistically significant or non-significant pooled result.

***Moderate evidence:*** statistically significant pooled results derived from multiple studies that are statistically heterogeneous, including at least one high quality study; or from multiple moderate quality or low quality studies which are statistically homogenous.

***Limited evidence:*** results from one high quality study or multiple moderate or low quality studies that are statistically heterogeneous.

***Very limited evidence:*** results from one moderate quality study or one low quality study.

***No evidence:*** pooled results insignificant and derived from multiple studies regardless of quality that are statistically heterogeneous.

## Results

### Search results

**Figure 1:**
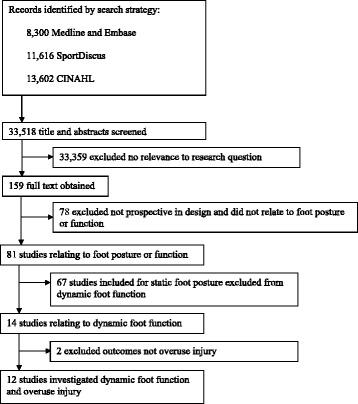
**Search results through the review process.**

Across the two parts of this systematic review (static foot posture and dynamic foot function), a total of 33,518 citations were retrieved from the electronic database search. Following the sequential review of titles, abstracts and full texts, as well as removing studies that were not prospective cohort studies, 80 studies were eligible (Figure 1). Of these, 12 studies investigated dynamic foot function variables, and were included in this part of the review [27,29,35,38-46]. Due to inconsistencies in outcomes measured, pooling of data was not possible.

### Quality assessment

Quality scores ranged from 0.44 to 1.20 (out of a possible total score of 2.00) (Additional file 3). With the exception of one moderate quality study [43], all studies were rated as low quality [27,29,35,37-42,44-46]. In terms of inter-rater reliability across 35 items included in the quality assessment, 24 items had perfect or near perfect agreement between raters. That is, these items were awarded the same score or there was a maximum of one point difference in scoring. For a further 10 items, the raters had near perfect agreement for 80% of the articles reviewed. Item 10 (‘reporting of adverse effects’) displayed the lowest agreement, with perfect or near perfect agreement for only 5/12 studies. Percentage agreement across the 35 items ranged from 17 to 100%.

All studies clearly reported the aim and objective (item 1) and that foot posture was measured prospectively before longer-term follow up of injury (item 28) [27,29,35,38-46]. Eleven studies clearly defined the assessment of foot function (item 2) [27,29,35,38-45] and eight studies clearly defined the lower limb overuse injury of interest (item 3) [29,35,39,41-45]. None of the included studies provided an adequate description of all intrinsic or extrinsic covariates or how these were adjusted for in the analysis (items 11, 12, 13, 36 and 37) (e.g. footwear worn, skill level or playing surface). Furthermore, no study provided an adequate report of the reliability and validity of foot function or injury outcome measurement of interest (items 25, 26, 31 and 32). Three studies provided an adequate standardisation procedure for assessing foot function (item 27) [39,42,45] and five studies reported standardisation of injury outcome (item 33).

Clear reporting of all data was present in four studies (items 14 and 15) [29,39,40,46]. However, the remaining seven studies primarily reported data only for significant relationships [27,35,38,42-45], while one study did not report any data [41]. Only one study reported effects for all results (odds or risk ratios) (item 16) [29]. With respect to generalisability of results, nine studies received a score of “Partial” (item 43) as results were deemed to be applicable to similar population groups to those investigated [29,35,38-45].

### Study characteristics

The 12 included studies incorporated a total of 3,773 participants. Table 1 presents a summary of study characteristics. The participant population varied, with five studies investigating military personal [27,29,39,41,43], five studies investigating runners [38,40,42,44,46], and two studies investigating cohorts of physical therapy students [35,45]. The types and incidence of lower limb overuse injuries reported were: tibial and femoral stress fractures, 8.7 to 10.0% [29,39]; iliotibial band syndrome, 9.4% [29,40]; patellofemoral pain, 4.0 to 17.0% [27,29,42-44]; medial tibial stress syndrome, 7.9% [41]; Achilles tendinopathy, 5.1 to 15.8% [29,44]; and non-specific lower limb overuse injuries, 14.0 to 20.6% [35,38,45].

**Table 1:** **Summary of study characteristics**

	**Population**	**Observation period (activity, duration)**	**Injury outcome**	**Injured group**		**Uninjured group**		**Gait assessment**	**Foot function measure**
				**N total (n females)**	**Age (mean ± SD)**	**N total (n females)**	**Age (mean ± SD)**		
Hesar et al., [38]	Athletics club members	3 running sessions/week; 10 weeks	LL overuse injury	27 (22)	41 ± 8	104 (89)	39 ± 11	Barefoot; 15 m runway; self-selected running speed	Plantar loading (Footscan)
Hetsroni et al., [27]	Military personal	4 month basic training course	Patellofemoral pain	NR	NR	NR	NR	Barefoot; treadmill running at 5 km/hr	Rearfoot kinematics (Ariel Dynamics Inc.)
Hetsroni et al., [39]	Military personal	4 month basic training course	Tibial and femoral stress fractures	Dependent on outcome variable investigated	NR	Dependent on outcome variable investigated	NR	Barefoot; treadmill running at 5 km/hr	Rearfoot kinematics (Ariel Dynamics Inc.)
Kaufman et al., [29]	Military personal	25 week training course	LL overuse injury	Dependent on outcome variable investigated	NR	Dependent on outcome variable investigated	NR	Boots and barefoot; self-selected walking speed (no mean or range presented)	Plantar pressure ratios – dynamic arch index (<4.14 cavus, >8.10 planus)
Noehren et al., [40]	Female runners	Individual non-specified running programs over a 2 year period	Iliotibial band syndrome	18 (18)	26	Dependent on outcome variable investigated	28	‘Standard running shoe’; running along a 25 runway at a speed of 3.7 m/s	Rearfoot kinematics (Vicon)
Noehren et al., [46]	Female runners	Individual non-specified running programs over a 2 year period	Patellofemoral pain	15 (15)	27 ± 10	15 (15)	27 ± 10	‘Standard running shoe’ (Nike, Pegasus); running along a 25 run way at a speed of 3.7 m/s	Rearfoot kinematics (Vicon)
Sharma et al., [41]	Male infantry recruits	26 week military training	Medial tibial stress syndrome	37 (0)	NR	239 (0)	NR	Barefoot; self selected walking speed (no mean or range presented)	Plantar loading (Footscan)
Thijs et al., [42]	Novice recreational runners	10 week start to run programme	Patellofemoral pain	17 (16)	39 ± 10	85 (NR)	37 ± 9	Barefoot; walking at a self-chosen, moderate velocity (no mean or range presented)	Plantar loading (Footscan)
Thijs et al., [43]	Military personal	6 week basic military training	Patellofemoral pain	36 (19)	19 ± 2	48 (NR)	19 ± 1	Barefoot; walking at a self-chosen, moderate velocity (no mean or range presented)	Plantar loading (Footscan)
Van Ginckel et al., [44]	Novice runners	10 week start to run programme	Achilles tendinopathy	10 (2)	38 ± 11	53 (45)	40 ± 9	Barefoot; self-selected jogging pace (no mean or range presented)	Plantar loading (Footscan)
Willems et al., [35]	Physical education students	University physical education course	LL overuse injury	46 (29)	NR	167 (NR)	NR	Barefoot; 3.3 m/s within a boundary of 0.17 m/s	Plantar loading (Footscan)/ankle, knee and hip kinematics and kinetics (Proreflex)
Willems et al., [45]	Physical education students	University physical education course	LL overuse injury	46 (29)	NR	167 (NR)	NR	‘Neutral running shoe’; 3.3 m/s within a boundary of 0.17 m/s	Plantar loading (Footscan)/ankle, knee and hip kinematics and kinetics (Proreflex)

LL = lower limb; NR = not reported.

Prior to prospective investigation, eight of the 12 studies investigated dynamic plantar loading (i.e. plantar pressure) [29,35,38,41-45], six investigated kinematic variables [27,35,39,40,45,46] and one investigated rearfoot joint moments [45] (Additional files 4 and 5). A large number of plantar pressure variables were evaluated. Baseline measures of foot function were commonly performed during unshod gait [27,29,35,38,39,41-44], although four studies obtained measures during shod gait [29,40,45,46]. Gait was assessed during treadmill walking at 5 kilometers per hour [27,39], or during overground walking or running at a self-selected speed [29,35,38,40-46]. Only four studies that investigated overground running reported mean values of the speed at which participants were observed, ranging between 3.3 to 3.7 metres per second [35,40,45,46].

### Dynamic foot function variables as risk factors for lower limb overuse injuries

**Figure 2:**
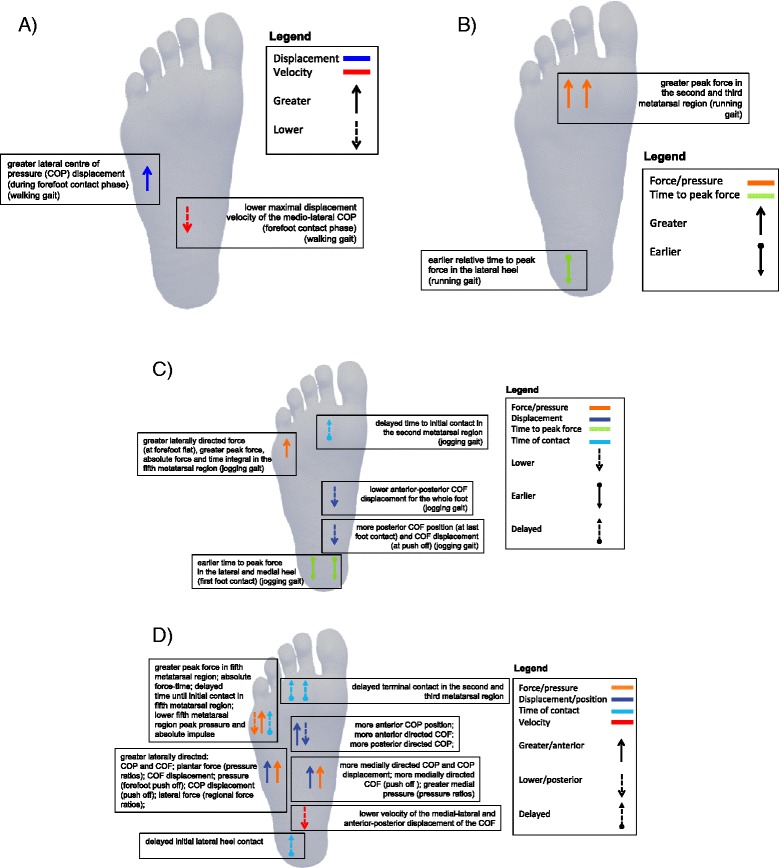
**Plantar pressure risk factors for: (A) patellofemoral pain (during walking); (B) patellofemoral pain (during running); (C) Achilles tendinopathy; and (D) non-specific injuries.** Force/pressure includes force time integral, impulse.

We found evidence supporting foot function as a risk factor for lower limb overuse injuries. There was *limited to very limited evidence* supporting (i) plantar loading and kinematic variables as risk factors for patellofemoral pain; (ii) plantar loading variables for Achilles tendinopathy; and (iii) plantar loading and kinematic variables for various non-specific lower limb overuse injuries. This is illustrated in Figure 2. For a complete reference of significant and non-significant findings for all injuries investigated, refer to Additional files 4 and 5.

### Patellofemoral pain

#### Plantar loading variables

There was *limited evidence* for plantar loading variables as a risk factor for patellofemoral pain, see Figures 2A and B. Participants who developed patellofemoral pain had earlier relative time to peak force in the lateral heel (SMD −0.56, 95% CI −1.09 to −0.37) and greater peak force in the second (0.65, 0.12 to 1.17) and third (0.60, 0.07 to 1.12) metatarsal regions during running [42]. Those who developed patellofemoral pain also demonstrated greater lateral centre of pressure (COP) displacement (−0.47, −0.90 to −0.03) and lower maximal displacement velocity of the mediolateral COP (−0.85, −1.29 to −0.39) during the ‘forefoot contact phase’ of walking [43].

#### Kinematic variables

**Figure 3:**
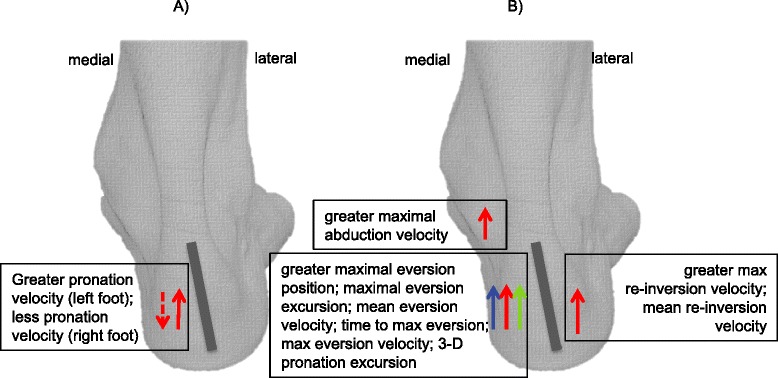
**Kinematic risk factors for: (A) patellofemoral pain; and (B) non-specific injuries.**

There was *very limited evidence* for kinematic variables as a risk factor for patellofemoral pain, see Figure 3A. A single study [27] investigated rearfoot kinematics, reporting opposite findings for the left and right sides. Greater pronation velocity on the left was a significant risk factor for patellofemoral pain development (quartile 4 versus quartile 3: RR 3.43 95% CI 1.32 to 8.96). Conversely, reduced pronation velocity of the right foot was a significant predictor of patellofemoral pain development (quartile 4 versus quartile 3: 0.38, 0.15 to 0.92). The authors did not specify whether the outcome (i.e. greater or reduced pronation velocity) was related to the side affected by patellofemoral pain.

### Achilles tendinopathy

#### Plantar loading variables

There was *very limited evidence* for plantar loading variables as a risk factor for mid-portion Achilles tendinopathy, evaluated in one study [44], see Figure 2C. Participants who developed Achilles tendinopathy exhibited significantly earlier time to peak force in the medial heel (SMD −0.716, 95% CI −1.39 to −0.02) and lateral heel (−1.08, −1.77 to −0.37), and delayed time to initial contact in the second metatarsal region (−1.00, −1.69 to −0.29). They also demonstrated greater peak force (0.84, 0.14 to 1.52) and a higher absolute force time integral (0.81, 0.11 to 1.49) in the fifth metatarsal region. In addition, those that developed Achilles tendinopathy displayed less anterior-posterior center of force (COF) displacement for the whole foot (−0.95, −1.64 to −0.25), greater laterally directed force in the forefoot at ‘forefoot flat’ (−0.88, −1.57 to −0.18) and a more posterior COF position at ‘last foot contact’ (−0.95, −1.63 to −0.24). During forefoot push-off, those that developed Achilles tendinopathy displayed more posterior COF displacement (−0.75, −1.43 to −0.05).

### Non-specific lower limb overuse injuries

There was *limited evidence* for plantar loading variables as a risk factor for non-specific lower limb overuse injuries, see Figure 2D.

#### Plantar loading variables - discrete plantar regions

Participants who developed a non-specific lower limb overuse injury exhibited delayed initial lateral heel contact (SMD 0.60, 95% CI 0.35 to 0.86) and terminal heel contact in the second and third metatarsal region (0.43, 0.18 to 0.68; 0.37, 0.12 to 0.62, respectively) [35]. In the fifth metatarsal region, an increase in peak force (0.52, 0.09 to 0.95 [38]) and absolute force-time integral (0.57, 0.14 to 1.00 [38]), as well as delayed time until initial contact (0.32, 0.07 to 0.57 [35]) were risk factors for non-specific overuse injury. However, contrary to these findings, Willems and colleagues reported lower fifth metatarsal region peak pressure (−0.44, −0.70 to −0.19) [35] and absolute impulse (−0.31, −0.56 to −0.05 [45]; −0.42, −0.67 to −0.17 [35]) in those who developed non-specific lower limb overuse injuries.

#### Plantar loading variables - time-specific gait events

At first foot contact, participants who developed a non-specific lower limb overuse injury had a more laterally directed COP (SMD −0.47, 95% CI −0.73 to −0.22) [45] and a more anterior COP position (0.31, 0.06 to 0.56) [35]. At first metatarsal contact, participants who developed a non-specific lower limb overuse injury had greater lateral force as indicated by three mediolateral regional force ratios (−0.55, −0.97 to −0.12; −0.57, −0.99 to −0.13; −0.59, −1.02 to −0.16) [38]. At forefoot flat, there was a lower velocity of the medio-lateral (−0.64, −1.07 to −0.21) and anterior-posterior displacement of the COF (−0.46, −0.88 to −0.03); and a more anterior COF position (0.61, 0.18 to 1.04) in those that developed non-specific lower limb overuse injuries [38]. Willems et al. [35,45] reported greater medial pressure as indicated by two pressure ratios (0.47, 0.22 to 0.72 [35]; 0.40, 0.09 to 0.59 [45]) and a more medially directed COP (0.38, 0.13 to 0.63) [35]. At heel-off, participants who developed a non-specific lower limb overuse injury had a more laterally directed COF (−0.70, −1.13 to −0.27) [38]. Contrary to this finding, Willems et al. [35,45] reported greater medial pressure, as indicated by two pressure ratios (0.33, 0.07 to 0.58 [35]; 0.33, 0.08 to 0.58 [45]) in those who developed overuse injuries. At last foot contact, participants who developed a non-specific overuse injury had a more laterally directed COP (−0.81, −1.07 to −0.55) [35], and more posterior COP position (−0.53, −0.79 to −0.28) [35].

#### Plantar loading variables (phase-specific gait events)

During the initial contact phase, participants who developed a non-specific lower limb overuse injury had a more laterally directed plantar force (SMD −0.43, 95% CI −0.85 to −0.001) [38]. Contrary to this finding, Willems et al. [45] reported a more medially directed pressure, as indicated by one pressure ratio (0.57, 0.31 to 0.82) and a more medially directed COP displacement (0.61, 0.36 to 0.86).

Hesar et al. [38] found that, during the forefoot contact phase, participants who developed a non-specific lower limb overuse injury had greater lateral COF displacement (−0.84, −1.27 to −0.40). Contrary to this finding, Willems et al. [35,45] reported a greater medial pressure (0.54, 0.29 to 0.79) [35] and a more medially directed COP displacement (0.58, 0.33 to 0.83 [35]; 0.31, 0.05 to 0.56 [45]).

Participants who developed a non-specific lower limb overuse injury had a more laterally directed COF displacement (−0.61, −1.03 to −0.17 [38]) during the foot flat phase, and a more medially directed COF during the forefoot push off phase (0.52, 0.09 to 0.94 [38]). Contrary to this latter finding, Willems et al. [35,45] reported a more laterally directed pressure during forefoot push off, as indicated by one pressure ratio (−0.35, −0.60 to −0.09) [45], and a more laterally directed COP displacement (−0.84, −1.09 to −0.58 [35]; −0.37, −0.62 to −0.12 [45]).

#### Kinematic variables

There was *limited evidence* for kinematic variables as a risk factor for non-specific lower limb overuse injuries, see Figure 3B. For the rearfoot segment, participants who developed a non-specific lower limb overuse injury exhibited a greater maximal eversion position (SMD 0.37, 95% CI 0.12 to 0.62) [35], eversion excursion (0.36, 0.10 to 0.61 [35]; 0.31, 0.06 to 0.56 [45]), mean eversion velocity (0.37, 0.12 to 0.62) [35], time to maximal eversion (0.39, 0.14 to 0.64) [45], maximal eversion velocity (0.39, 0.14 to 0.64 [35]; 0.29, 0.03 to 0.54 [45]), mean inversion velocity (0.44, 0.18 to 0.69) [35], maximal re-inversion velocity (0.41, 0.16 to 0.66) [45], and mean re-inversion velocity (0.31, 0.06 to 0.56) [45].

In the forefoot segment, participants who developed a non-specific lower limb overuse injury exhibited greater maximal abduction velocity (0.62, 0.37 to 0.88) [35] and abduction excursion (0.36, 0.10 to 0.61 [35]; 0.31, 0.06 to 0.56 [45]). One study derived a three-dimensional pronation angle from eversion, abduction and dorsiflexion excursions, and reported that participants who developed a non-specific lower limb overuse injury exhibited greater three-dimensional pronation excursion (0.49, 0.23 to 0.74) [45].

### Other lower limb overuse injuries

There was no evidence supporting dynamic foot function as a risk factor for any other lower limb overuse injury. Non-significant effects were found for iliotibial band syndrome [29,40] and stress fractures [29].

## Discussion

This systematic review evaluated current evidence for dynamic foot function as a risk factor for the development of lower limb overuse injuries. From six of the twelve studies included, we found very limited evidence that plantar pressure and kinematic variables representing dynamic foot function are associated with an increased risk of patellofemoral pain, Achilles tendinopathy and non-specific lower limb overuse injury [35,38,42-45]. Notably, significant findings reported across the studies had small to moderate effect sizes, and many 95% confidence intervals included zero, indicating non-significant findings.

Plantar pressure patterns associated with patellofemoral pain differed for walking and running gait. Risk factors in walking gait included greater lateral COP displacement and lower maximal displacement of the medio-lateral COP [42], whereas for running gait risk factors included earlier time to peak force in the lateral heel and greater peak force in the second and third metatarsal region [43]. While it is difficult to suggest a mechanism linking these plantar pressure differences with the development of patellofemoral pain, Thijs et al. [42,43] speculated that these findings may indicate a resultant reduction in foot pronation during the loading phase of gait, and subsequent reduction in shock attenuation at the foot. This could increase transfer of ground reaction forces to more proximal structures, such as the patellofemoral joint.

Plantar pressure patterns associated with Achilles tendinopathy were evident from one study investigating jogging gait, and included earlier time to peak force in the lateral heel, less posterior COF displacement/more posterior COF position, greater laterally directed force and delayed time to initial contact in the second metatarsal region [44]. Van Ginkel and colleagues [44] speculated that these findings may indicate a more lateral foot roll-over following heel strike and diminished forward force transfer from the rearfoot to the forefoot. It is plausible that differences in force transfer across the foot may lead to altered loading of the Achilles tendon and contribute to injury, but this requires further evaluation.

Another consideration is that increased lateral loading at the foot is an adaptive response to proximal mechanics that increase medial lower limb loading. Prospective studies have shown that increased hip adduction during overground running [46] and increased hip internal rotation when landing from a drop jump [22] are risk factors for the development of patellofemoral pain. Furthermore, cross-sectional studies have reported deficits in neuromuscular control of the hip in those with patellofemoral pain [55-61] and Achilles tendinopathy [62,63]. Further research is required to better understand the relationship between proximal and distal mechanics during gait, and risk of overuse injury development.

In contrast to evidence we found regarding plantar pressure, we found very few kinematic risk factors for lower limb overuse injuries. Our search strategy identified only one study that investigated kinematic risk factors for patellofemoral pain, which presented contradictory findings, no prospective studies that investigated kinematic risk factors for Achilles tendinopathy and two studies that reported differences in rearfoot eversion and forefoot abduction as risk factors for non-specific injuries [35,45]. Whilst cross-sectional findings indicate differences in foot kinematics in people with patellofemoral pain [64] and Achilles tendinopathy [49], we found a lack of prospective kinematic data to indicate the temporal relationship between foot kinematics and overuse injury. Thus, at this time it is difficult to draw conclusions as to whether altered foot kinematics is a clear risk factor for lower limb overuse injuries.

In addition to necessitating more kinematic studies, consideration needs to be given to the method of measuring foot kinematics. Considering that overuse injuries generally involve cumulative exposure to load, it is plausible that those who develop overuse injuries demonstrate subtle kinematic differences that are not detectable by current kinematic measures. This is supported by previous findings regarding a lack of biomechanical coupling of plantar pressure indices and angular movements recorded between the calcaneus and the tibia [65]. Further studies are required to increase understanding of this relationship, which could be achieved using more sophisticated three-dimensional and multisegment foot modeling techniques, and more clinically applicable measures of foot function.

Not surprisingly, it was difficult to identify a systematic pattern of plantar loading and kinematic risk factors for the category of ‘non-specific injuries’. For example, significant risk factors were evident for greater lateral and medial directed COP, as well as increases and decreases in pressure-related outcomes in the fifth metatarsal region. While these findings indeed add evidence of a relationship between dynamic foot function and lower limb injury, the nature of the relationship is unpredictable, and likely relates to the variability of injuries evaluated under the term ‘non-specific injuries’. Therefore, with the advancement and availability of diagnostic algorithms and imaging for lower limb injury, future research should avoid pooling all injuries, and instead focus efforts on exploring conditions that are discrete and well-defined. This is likely to enhance identification of injury-specific risk factors.

Interestingly, we found no evidence that dynamic foot function is a risk factor for iliotibial band syndrome or lower limb stress fractures including the foot. Findings from Noehren et al. [46] indicated that aberrant hip mechanics may be a stronger risk factor for iliotibial band syndrome than dynamic foot function. They reported that increased hip adduction during running, but not rearfoot eversion, was a predictor of patellofemoral pain development in a cohort of 400 female runners [46]. This is logical given the proposed mechanism of iliotibial band syndrome, where increased tension on the iliotibial band compresses the lateral femoral epicondyle [46].

The lack of foot specific injuries (e.g. plantar fasciitis, metatarsal stress fracture) associated with dynamic foot function is another unexpected finding. Although Kaufman et al. [29] reported that dynamic pes planus in shoes, measured as the ratio of midfoot contact area to total contact area, was a significant predictor of lower limb stress fracture (one third of which involved the foot), our effect size calculations were not significant. This is because the authors set significance at 0.10, whereas we used the more conventional alpha of 0.05. Because of the large number of variables evaluated, this is the more conservative approach to reduce the risk of type II error. An earlier study also reported that pronated foot type (i.e. static foot posture) was a significant risk factor for metatarsal stress fractures, while a supinated foot type was a risk factor for tibial and femoral stress fractures [66]. However, the static x-ray measure of foot type used in this study may not correlate with dynamic foot function. It is plausible that lower limb stress fractures are more a function of bony overload due to the application of external loads, rather than the biomechanical characteristics of the foot. This is in part supported by the use of military cohorts in both studies [29,66]. The influence of dynamic foot function on the development of lower limb stress fractures should be investigated in civilian populations to ascertain this.

Plantar loading variables were the most abundant risk factor identified for lower limb injury, albeit a relatively low risk with small to moderate effect sizes. In terms of the clinical application of these findings, it is difficult to map the plantar pressure risk factors to specific static foot types. De Cock et al. [67] reported that participants with low arched feet had a more laterally directed COP across the gait cycle. This is consistent with our plantar pressure findings relating to patellofemoral pain and Achilles tendinopathy. Conversely, Wong et al. [68] investigated the effect of foot morphology on center-of-pressure excursion during barefoot walking. Their findings indicated that more supinated foot types displayed a larger area of lateral COP excursion, and, conversely, more pronated foot types displayed a smaller area of lateral COP excursion. However, these findings were taken over the entire gait cycle, rather than the discrete phases evaluated in the prospective studies included in this review. In light of the volume of studies that use plantar pressure measures to evaluate dynamic foot function, there is a clear need for further studies to investigate methods of transferring plantar pressure information to clinically relevant measures.

Nevertheless, having some limited knowledge of the pattern of plantar loading risk factors may serve to inform the design of new and existing interventions that may redistribute or counter-balance plantar loading patterns observed in people at risk of injury. For example, arch-contoured foot orthoses alter plantar pressure systematically by reducing pressure in the forefoot and heel regions, and redistributing pressure to the midfoot [69]. With this in mind, there is evidence from pooled data from randomised clinical trials (RCTs) that foot orthoses are effective in preventing lower limb overuse injuries [70], as well as evidence from high-quality RCTs that foot orthoses reduce symptoms associated with patellofemoral pain [71]. In the absence of evidence regarding kinematic effects, our findings suggest that foot orthoses may exert their clinical effects by redistributing plantar pressure (i.e. alter the magnitude, location and temporal patterns of reaction forces at the foot-orthosis interface). However, this requires further investigation.

Whilst this review has highlighted specific measures of dynamic foot function that are risk factors for lower limb overuse injuries, there are several limitations to the identification of these risk factors in a clinical practice setting. Firstly, while findings indicate the direction of altered plantar loading that may increase the risk of development of Achilles tendinopathy or patellofemoral pain, there are no reported thresholds of when an individual is deemed at risk (e.g. peak force in forefoot region exceeding 150 N). Future investigations are required to establish clinical guidelines and screening criteria for these risk factors. Secondly, the assessment of plantar pressures and three-dimensional kinematics requires expensive and sophisticated equipment that is not readily available in clinical practice settings, as well as specialised training in performing and processing these measurements. Future studies should investigate the translation of these laboratory-based measures to clinically applicable measures.

There are also limitations associated with the included studies. The majority of studies evaluated foot function while walking or running barefoot, which may limit the generalisability of findings to shod gait. While it is acknowledged that there are limitations associated with measuring plantar pressures and kinematics while wearing shoes, this is the condition that most closely resembles gait during daily and sporting activities. There were also differences between studies in the evaluation of overground versus treadmill gait analysis. As different gait patterns have been observed for treadmill and overground gait [72,73], it may be inappropriate to measure dynamic foot function during treadmill gait in habitual overground runners, and vice versa. This may lead to a discrepancy between dynamic foot function measured during testing, and foot function during cumulative usual activity. A further limitation of this systematic review is that the methodological quality of the majority of included studies was generally poor. This was largely related to inadequate reporting of foot function measures, covariates, and non-significant results. Thus, the findings should be considered with this in mind. In order to enhance the overall quality of research in this field, future prospective studies should comply with published guidelines for minimum standards of reporting [74].

## Conclusion

This systematic review identified very limited evidence, with small to moderate effect sizes, that dynamic foot function during walking and running is a risk factor for patellofemoral pain, Achilles tendinopathy, and non-specific lower limb overuse injuries. More lateral plantar loading patterns were found to be risk factors for patellofemoral pain and Achilles tendinopathy. Findings from three studies indicate that there is no evidence that dynamic foot function is a risk factor for iliotibial band syndrome or lower limb stress fractures. At present, it is unclear whether these risk factors can be identified clinically (without sophisticated equipment), or modified to prevent or manage overuse injuries. Future prospective studies should address methodological limitations, avoid grouping different lower limb injuries in analyses, and explore clinically meaningful representations of dynamic foot function.

## Additional files

Additional file 1:
**Search strategy.**
Additional file 2:
**Epidemiological Appraisal Instrument used to rate the quality of the 12 included studies.**
Additional file 3:
**Results from quality assessment using the Epidemiological Appraisal.** Instrument (12 included studies). Additional file 4:
**Presentation of plantar loading variables across the 12 studies.**
Additional file 5:
**Presentation of kinematic and kinetic variables across the 12 studies.**
