# Supplementary material for: Dynamic foot function as a risk factor for lower limb overuse injury: a systematic review
Source: J Foot Ankle Res. 2014 Dec 19;7:53. doi: 10.1186/s13047-014-0053-6 (PMC4296532; doi:10.1186/s13047-014-0053-6)
Supplement: Additional file 3: — Results from quality assessment using the Epidemiological Appraisal. Instrument (12 included studies). [file 13047_2014_53_MOESM3_ESM.pdf]

Additional file 3 - Results from quality assessment using the Epidemiological Appraisal Instrument (12 studies included)

Black shading = “Yes”, Grey shading = “Partial”, White (no shading) = “No” or “Unable to determine”, “-” = “Not applicable”

Study ID	Hesar et al., 2009	Hetsroni et al., 2006	Hetsroni et al., 2008	Kaufman et al., 1999	Noehren et al., 2007	Noehren et al., 2012	Sharma et al., 2012	Thijs et al., 2007	Thijs et al., 2008	Van Ginckel et al., 2009	Willems et al., 2006	Willems et al., 2007	Studies scoring yes (x/12)
Q1. Reported study aim/objective clearly	■	■	■	■	■	■	■	■	■	■	■	■	12
Q2. Risk factors related to foot posture are clearly defined	■	■	■	■	■	▒	■	■	■	■	■	■	11
Q3. Overuse injury clearly defined		▒	■	■	▒	▒	■	■	■	■	■	■	8
Q4. Reported study design	■	▒	▒	▒	▒	▒	▒	■	■	■	■	▒	5
Q5. Reported subject population	▒	▒	▒	■	▒		▒	■	▒	▒	▒	▒	2
Q6. Reported inclusion criteria	■		▒		■	▒	▒	■	■	▒	■	■	6
Q7. Reported participation rate				■				■	■				3
Q8. Reported participant characteristics	■		▒	■	▒		■	■	■	■	■	■	8
Q9. Reported participant lost characteristics				▒				■					1
Q10. Reported adverse reactions	-	-	-	-	-	-	-	-	-	-	-	-	-
Q11. Reported intrinsic risk factors	▒		▒	▒	▒	▒	▒	▒	▒	▒	▒	▒	0
Q12. Reported extrinsic risk factors	▒	▒	▒	▒		▒	▒	▒	▒	▒	▒	▒	0
Q13. Reported statistical methods	▒						▒	▒	▒	▒			0
Q14. Reported all basic data	▒	▒	■	■	■	■		▒	▒	■	▒	▒	5
Q15. Reported variability in data	▒	▒	■	■	■	■		▒	▒	■	▒	▒	5
Q16. Reported statistical parameters	▒		▒	■			▒			▒			1
Q17. Sample size calculations		▒			▒	▒							0
Q18. Comparability of case/control groups	-	-	-	-	-	-	-	-	-	-	-	-	-
Q19. Adequate participation rate				■				■	■				3
Q20. Recruitment period								■					1
Q21. Non-responder characteristics described													0
Q22. New incident case analysis	-	-	-	-	-	-	-	-	-	-	-	-	-
Q23. Subject randomisation	-	-	-	-	-	-	-	-	-	-	-	-	-
Q24. Randomisation concealment	-	-	-	-	-	-	-	-	-	-	-	-	-
Q25. Reported reliablity of foot posture risk factor measurement						▒							0
Q26. Reported validity of foot posture risk factor measurement													0
Q27. Reported standardisation of foot posture risk factor measurement			■		▒	▒		▒	■			■	3
Q28. Was foot posture risk factor measurement conducted prior to injury	■	■	■	■	■	■	■	■	■	■	■	■	12
Q29. Observer blinding	-	-		-	-	-	-	-	-	-	-	-	0
Q30. Subject blinding	-	-		-	-	-	-	-	-	-	-	-	0

Q31. Reported reliablity of overuse injury measurement													0
Q32. Reported validity of overuse injury measurement													0
Q33. Reported standardisation of overuse injury measurement													5
Q34. Observation time comparability													9
Q35. Reported prior disease/history	-				-	-		-	-	-	-	-	0
Q36. Adjustment for intrinsic variables													0
Q37. Adjustment for extrinsic variables													0
Q38. Is follow up time adequate													6
Q39. Follow up time differences													9
Q40. Reported data for ≥3 levels of associated factors													6
Q41. Reported data for subgroups of subjects (e.g. by gender or age)													4
Q42. Generalisability of results to study population (participation rate)													0
Q43. Generalisability of results to other populations (random sampling)													0
Overall quality score (range 0 to 2)	0.83	0.44	0.84	1.03	0.8	0.71	0.69	1.20	1.09	0.97	0.74	0.74	

Good article (1.40 SD +0.30), average article (1.33 SD +23) or poor article (0.90 SD ± 18) from Genaidy et al. [46]
