# Supplementary material for: Dynamic foot function as a risk factor for lower limb overuse injury: a systematic review
Source: J Foot Ankle Res. 2014 Dec 19;7:53. doi: 10.1186/s13047-014-0053-6 (PMC4296532; doi:10.1186/s13047-014-0053-6)
Supplement: Additional file 4: — Presentation of plantar loading variables across the 12 studies. [file 13047_2014_53_MOESM4_ESM.docx]

**Additional file 4 – Plantar pressure analysis**

Patellofemoral pain continuous plantar variables

|  | Variable | | Region | Study ID | Effect size (ES) | Confidence interval | |  |
| --- | --- | --- | --- | --- | --- | --- | --- | --- |
|  | | |  |  |  | Lower | Upper |  |
| **Plantar regions** | | | | | | | |  |
| Whole foot | | Total foot contact time |  | Thijs et al., 2008 | NR | | |  |
| Medial heel  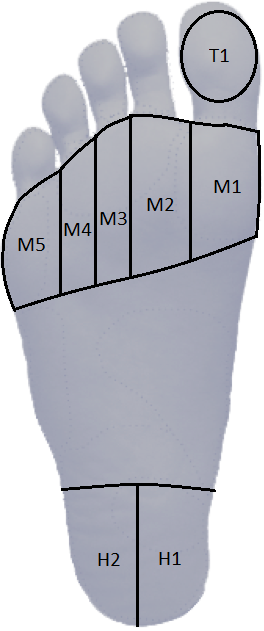  Adapted from Hesar et al. [38] | | Peak force | H1 | Thijs et al., 2008 | 0.333 | -0.192 | 0.854 |  |
|  |  | Absolute force-time integral | H1 | Thijs et al., 2008 | NR | | |  |
|  |  | Relative force-time integral | H1 | Thijs et al., 2008 | NR | | |  |
|  |  | Relative Time to peak force | H1 | Thijs et al., 2008 | -0.467 | -0.988 | 0.061 |  |
|  |  | Peak Pressure | H1 | Thijs et al., 2007 | NR | | |  |
|  |  | Absolute Impulses | H1 | Thijs et al., 2007 | NR | | |  |
|  |  | Relative Impulses | H1 | Thijs et al., 2007 | NR | | |  |
|  |  | Time to peak pressure | H1 | Thijs et al., 2007 | 0.116 | -0.317 | 0.548 |  |
|  |  | Time at which region made contact | H1 | Thijs et al., 2007 | NR | | |  |
|  |  |  | H1 | Thijs et al., 2008 | NR | | |  |
|  |  | Time at which the region ended contact | H1 | Thijs et al., 2007 | -0.060 | -0.492 | 0.372 |  |
|  |  |  | H1 | Thijs et al., 2008 | NR | | |  |
| Lateral heel  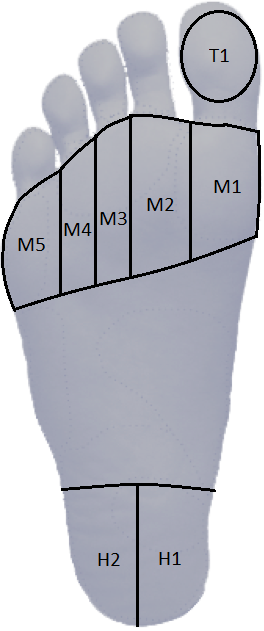  Adapted from Hesar et al. [38] | | Peak force | H2 | Thijs et al., 2008 | 0.499 | -0.030 | 1.020 |  |
|  |  | Absolute force-time integral | H2 | Thijs et al., 2008 | NR | | |  |
|  |  | Relative force-time integral | H2 | Thijs et al., 2008 | NR | | |  |
|  |  | Relative time to peak force | H2 | Thijs et al., 2008 | -0.562 | -1.085 | -0.032 |  |
|  |  | Peak Pressure | H2 | Thijs et al., 2007 | NR | | |  |
|  |  | Absolute Impulses | H2 | Thijs et al., 2007 | NR | | |  |
|  |  | Relative Impulses | H2 | Thijs et al., 2007 | NR | | |  |
|  |  | Time to peak pressure | H2 | Thijs et al., 2007 | -0.149 | -0.581 | 0.285 |  |
|  |  | Time at which region made contact | H2 | Thijs et al., 2007 | NR | | |  |
|  |  |  | H2 | Thijs et al., 2008 | NR | | |  |
|  |  | Time at which the region ended contact | H2 | Thijs et al., 2007 | -0.108 | -0.539 | 0.326 |  |
|  |  |  | H2 | Thijs et al., 2008 | NR | | |  |

| Medial forefoot (M1-M3)  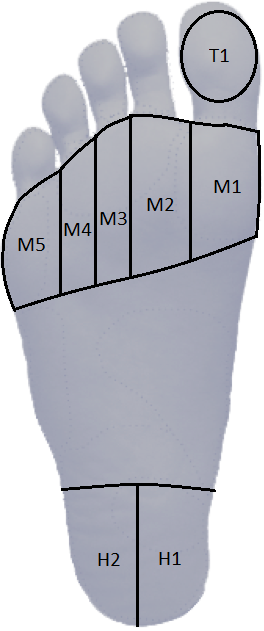  Adapted from Hesar et al. [38] | Peak force | M1 | Thijs et al., 2008 | 0.263 | -0.261 | 0.783 |
| --- | --- | --- | --- | --- | --- | --- |
|  |  | M2 |  | 0.650 | 0.117 | 1.174 |
|  |  | M3 |  | 0.600 | 0.069 | 1.123 |
|  | Absolute force-time integral | M1 | Thijs et al., 2008 | NR | | |
|  |  | M2 |  | NR | | |
|  |  | M3 |  | NR | | |
|  | Relative force-time integral | M1 | Thijs et al., 2008 | NR | | |
|  |  | M2 |  | NR | | |
|  |  | M3 |  | NR | | |
|  | Relative Time to peak force | M1 | Thijs et al., 2008 | -0.310 | -0.831 | 0.214 |
|  |  | M2 |  | 0.100 | -0.422 | 0.620 |
|  |  | M3 |  | -0.077 | -0.597 | 0.445 |
|  | Peak Pressure | M1 | Thijs et al., 2007 | NR | | |
|  |  | M2 |  | NR | | |
|  |  | M3 |  | NR | | |
|  | Absolute Impulses | M1 | Thijs et al., 2007 | NR | | |
|  |  | M2 |  | NR | | |
|  |  | M3 |  | NR | | |
|  | Relative Impulses | M1 | Thijs et al., 2007 | NR | | |
|  |  | M2 |  | NR | | |
|  |  | M3 |  | NR | | |
|  | Time to peak pressure | M1 | Thijs et al., 2007 | -0.105 | -0.536 | 0.329 |
|  |  | M2 |  | -0.045 | -0.477 | 0.387 |
|  |  | M3 |  | -0.187 | -0.618 | 0.248 |
|  | Time at which region made contact | M1 | Thijs et al., 2007 | -0.237 | -0.668 | 0.199 |
|  |  | M2 |  | 0.000 | -0.432 | 0.432 |
|  |  | M3 |  | 0.118 | -0.316 | 0.549 |
|  |  | M1 | Thijs et al., 2008 | NR | | |
|  |  | M2 |  | NR | | |
|  |  | M3 |  | NR | | |
|  | Time at which the region ended contact | M1 | Thijs et al., 2007 | 0.170 | -0.265 | 0.601 |
|  |  | M2 |  | 0.132 | -0.302 | 0.563 |
|  |  | M3 |  | 0.088 | -0.345 | 0.519 |
|  |  | M1 | Thijs et al., 2008 | NR | | |
|  |  | M2 |  | NR | | |
|  |  | M3 |  | NR | | |

| Lateral forefoot (M4 and M5)  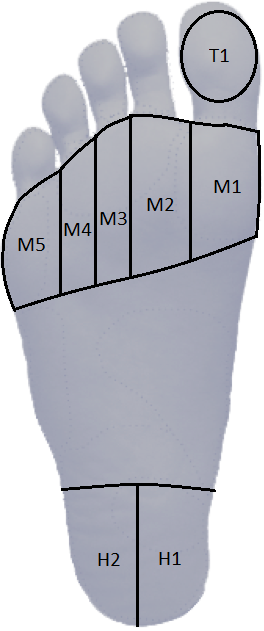  Adapted from Hesar et al. [38] | Peak force | M4 | Thijs et al., 2008 | 0.509 | -0.020 | 1.031 |
| --- | --- | --- | --- | --- | --- | --- |
|  |  | M5 |  | 0.456 | -0.072 | 0.977 |
|  | Absolute force-time integral | M4 | Thijs et al., 2008 | NR | | |
|  |  | M5 |  | NR | | |
|  | Relative force-time integral | M4 | Thijs et al., 2008 | NR | | |
|  |  | M5 |  | NR | | |
|  | Relative Time to peak force | M4 | Thijs et al., 2008 | 0.250 | -0.273 | 0.770 |
|  |  | M5 |  | 0.347 | -0.178 | 0.867 |
|  | Peak pressure | M4 | Thijs et al., 2007 | NR | | |
|  |  | M5 |  | NR | | |
|  | Absolute Impulses | M4 | Thijs et al., 2007 | NR | | |
|  |  | M5 |  | NR | | |
|  | Relative Impulses | M4 | Thijs et al., 2007 | NR | | |
|  |  | M5 |  | NR | | |
|  | Time to peak pressure | M4 | Thijs et al., 2007 | -0.440 | -0.872 | 0.002 |
|  |  | M5 |  | -0.272 | -0.703 | 0.165 |
|  | Time at which region made contact | M4 | Thijs et al., 2007 | 0.344 | -0.094 | 0.776 |
|  |  | M5 |  | 0.106 | -0.328 | 0.537 |
|  |  | M4 | Thijs et al., 2008 | NR | | |
|  |  | M5 |  | NR | | |
|  | Time at which the region ended contact | M4 | Thijs et al., 2007 | 0.045 | -0.387 | 0.477 |
|  |  | M5 |  | -0.084 | -0.516 | 0.349 |
|  |  | M4 | Thijs et al., 2008 | NR | | |
|  |  | M5 |  | NR | | |
| Hallux (T1)  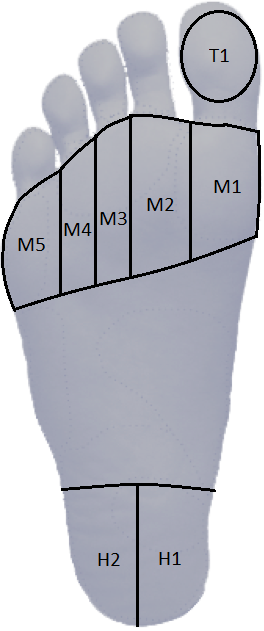  Adapted from Hesar et al. [38] | Peak Force | T1 | Thijs et al., 2008 | 0.257 | -0.267 | 0.777 |
|  | Absolute force-time integral | T1 | Thijs et al., 2008 | NR | | |
|  | Relative force-time integral | T1 | Thijs et al., 2008 | NR | | |
|  | Relative Time to peak force | T1 | Thijs et al., 2008 | 0.065 | -0.457 | 0.585 |
|  | Peak pressure | T1 | Thijs et al., 2007 | NR | | |
|  | Absolute Impulses | T1 | Thijs et al., 2007 | NR | | |
|  | Relative Impulses | T1 | Thijs et al., 2007 | NR | | |
|  | Time to peak Pressure | T1 | Thijs et al., 2007 | -0.045 | -0.477 | 0.387 |
|  | Time at which region made contact | T1 | Thijs et al., 2007 | -0.034 | -0.466 | 0.399 |
|  |  | T1 | Thijs et al., 2008 | NR | | |
|  | Time at which the region ended contact | T1 | Thijs et al., 2007 | -0.081 | -0.512 | 0.352 |
|  |  | T1 | Thijs et al., 2008 | NR | | |

| **Time specific variables during stance phase** | | | | | | |
| --- | --- | --- | --- | --- | --- | --- |
| FFC (first foot contact) | Force ratios | Mediolateral force ratio  (M1 + M2 + H1)/(M4 + M5 + H2) | Thijs et al., 2008 | NR | | |
|  | COF mediolateral Component (position) |  | Thijs et al., 2008 | NR | | |
|  | COF anterior posterior component (position) |  | Thijs et al., 2008 | NR | | |
|  | Pressure ratios | Mediolateral pressure ratio  (M1 + M2 + H1)/(M4 + M5 + H2) | Thijs et al., 2007 | -0.363 | -0.795 | 0.076 |
|  | COP mediolateral component (position) |  | Thijs et al., 2007 | NR | | |
|  | COP Velocity Mediolateral Component |  | Thijs et al., 2007 | NR | | |
|  | COP Anterior posterior Component (position) |  | Thijs et al., 2007 | NR | | |
|  | COP Velocity Anterior posterior Component |  | Thijs et al., 2007 | NR | | |
| FMC (first metatarsal contact) | Force ratios | Mediolateral force ratio  (M1 + M2 + H1)/(M4 + M5 + H2) | Thijs et al., 2008 | NR | | |
|  | COF mediolateral component (position) |  | Thijs et al., 2008 | NR | | |
|  | COF anterior- posterior component (position) |  | Thijs et al., 2008 | NR | | |
|  | Pressure ratios | Mediolateral pressure ratio  (M1 + M2 + H1)/(M4 + M5 + H2) | Thijs et al., 2007 | 0.070 | -0.362 | 0.502 |
|  | COP mediolateral component (position) |  | Thijs et al., 2007 | NR | | |
|  | COP velocity mediolateral component |  | Thijs et al., 2007 | NR | | |
|  | COP anterior- posterior component (position) |  | Thijs et al., 2007 | NR | | |
|  | COP velocity anterior posterior component |  | Thijs et al., 2007 | NR | | |
| FFF (forefoot flat) | Force ratios | Mediolateral force ratio  (M1 + M2 + H1)/(M4 + M5 + H2) | Thijs et al., 2008 | NR | | |
|  | COF mediolateral component (position) |  | Thijs et al., 2008 | NR | | |
|  | COF anterior posterior component (position) |  | Thijs et al., 2008 | NR | | |
|  | Pressure ratios | Mediolateral pressure ratio  (M1 + M2 + H1)/(M4 + M5 + H2) Medial foot / Lateral foot | Thijs et al., 2007 | 0.199 | -0.236 | 0.631 |
|  | COP mediolateral component (position) |  | Thijs et al., 2007 | NR | | |
|  | COP velocity mediolateral component |  | Thijs et al., 2007 | NR | | |
|  | COP anterior posterior component (position) |  | Thijs et al., 2007 | NR | | |
|  | COP velocity anterior posterior component |  | Thijs et al., 2007 | NR | | |
| HO (heel off) | Force ratios | Mediolateral force ratio  (M1 + M2 + H1)/(M4 + M5 + H2) | Thijs et al., 2008 | NR | | |
|  | COF mediolateral component (position) |  | Thijs et al., 2008 | NR | | |
|  | COF anterior posterior component (position) |  | Thijs et al., 2008 | NR | | |
|  | Pressure ratios | Mediolateral Pressure ratio  (M1 + M2 + H1)/(M4 + M5 + H2) | Thijs et al., 2007 | 0.211 | -0.225 | 0.642 |
|  | COP mediolateral component (position) |  | Thijs et al., 2007 | NR | | |
|  | COP velocity mediolateral component |  | Thijs et al., 2007 | NR | | |
|  | COP anterior posterior Component (position) |  | Thijs et al., 2007 | NR | | |
|  | COP velocity anterior posterior component |  | Thijs et al., 2007 | NR | | |
| LFC (last foot contact) | Force ratios | Mediolateral Force Ratio  (M1 + M2 + H1)/(M4 + M5 + H2) | Thijs et al., 2008 | NR | | |
|  | COF mediolateral component (position) |  | Thijs et al., 2008 | NR | | |
|  | COF anterior posterior component (position) |  | Thijs et al., 2008 | NR | | |
|  | Pressure ratios | Mediolateral Pressure ratio  (M1 + M2 + H1)/(M4 + M5 + H2) | Thijs et al., 2007 | -0.185 | -0.616 | 0.250 |
|  | COP mediolateral component (position) |  | Thijs et al., 2007 | NR | | |
|  | COP velocity mediolateral Component |  | Thijs et al., 2007 | NR | | |
|  | COP anterior posterior component (position) |  | Thijs et al., 2007 | NR | | |
|  | COP velocity anterior posterior component |  | Thijs et al., 2007 | NR | | |

| **Phase specific variables during stance phase** | | | | | | |
| --- | --- | --- | --- | --- | --- | --- |
| ICP initial contact phase | Force ratios | Mediolateral force ratio  (M1 + M2 + H1)/(M4 + M5 + H2) | Thijs et al., 2008 | NR | | |
|  | COF mediolateral component (displacement) |  | Thijs et al., 2008 | NR | | |
|  | COF anterior posterior component (displacement) |  | Thijs et al., 2008 | NR | | |
|  | Pressure ratios | Mediolateral pressure ratio  (M1 + M2 + H1)/(M4 + M5 + H2) | Thijs et al., 2007 | NR | | |
|  | COP mediolateral component (displacement) |  | Thijs et al., 2007 | -0.030 | -0.462 | 0.403 |
|  | COP velocity mediolateral component (displacement) |  | Thijs et al., 2007 | NR | | |
|  | COP maximal velocity mediolateral component (displacement) |  | Thijs et al., 2007 | 0.103 | -0.330 | 0.535 |
|  | COP anterior posterior component (displacement) |  | Thijs et al., 2007 | NR | | |
|  | COP velocity anterior posterior component (displacement) |  | Thijs et al., 2007 | NR | | |
|  | COP maximal velocity anterior posterior component (displacement) |  | Thijs et al., 2007 | NR | | |
| FFCP (forefoot contract phase) | Force ratios | Mediolateral force ratio  (M1 + M2 + H1)/(M4 + M5 + H2) | Thijs et al., 2008 | NR | | |
|  | COF mediolateral component (displacement) |  | Thijs et al., 2008 | NR | | |
|  | COF anterior posterior component (displacement) |  | Thijs et al., 2008 | NR | | |
|  | Pressure ratios | Mediolateral pressure ratio  (M1 + M2 + H1)/(M4 + M5 + H2) | Thijs et al., 2007 | NR | | |
|  | COP mediolateral component (displacement) |  | Thijs et al., 2007 | -0.470 | -0.903 | -0.028 |
|  | COP velocity mediolateral component (displacement) |  | Thijs et al., 2007 | NR | | |
|  | COP maximal velocity mediolateral component (displacement) |  | Thijs et al., 2007 | -0.845 | -1.287 | -0.387 |
|  | COP anterior posterior component (displacement) |  | Thijs et al., 2007 | NR | | |
|  | COP velocity anterior posterior component (displacement) |  | Thijs et al., 2007 | NR | | |
|  | COP maximal velocity anterior posterior component (displacement) |  | Thijs et al., 2007 | NR | | |
| FFP (foot flat phase) | Force ratios | Mediolateral force ratio  (M1 + M2 + H1)/(M4 + M5 + H2) | Thijs et al., 2008 | NR | | |
|  | COF mediolateral component (displacement) |  | Thijs et al., 2008 | NR | | |
|  | COF anterior posterior component (displacement) |  | Thijs et al., 2008 | NR | | |
|  | Pressure ratios | Mediolateral pressure ratio  (M1 + M2 + H1)/(M4 + M5 + H2) | Thijs et al., 2007 | NR | | |
|  | COP mediolateral component (displacement) |  | Thijs et al., 2007 | -0.043 | -0.475 | 0.390 |
|  | COP velocity mediolateral component (displacement) |  | Thijs et al., 2007 | NR | | |
|  | COP maximal velocity mediolateral component (displacement) |  | Thijs et al., 2007 | -0.112 | -0.543 | 0.322 |
|  | COP anterior posterior component (displacement) |  | Thijs et al., 2007 | NR | | |
|  | COP velocity anterior posterior component (displacement) |  | Thijs et al., 2007 | NR | | |
|  | COP maximal velocity anterior posterior component (displacement) |  | Thijs et al., 2007 | NR | | |
| FFPOP (forefoot push off phase) | Force ratios | Mediolateral force ratio  (M1 + M2 + H1)/(M4 + M5 + H2) | Thijs et al., 2008 | NR | | |
|  | COF mediolateral component (displacement) |  | Thijs et al., 2008 | NR | | |
|  | COF anterior posterior component (displacement) |  | Thijs et al., 2008 | NR | | |
|  | Pressure ratios | Mediolateral pressure ratio  (M1 + M2 + H1)/(M4 + M5 + H2) | Thijs et al., 2007 | NR | | |
|  | COP mediolateral component (displacement) |  | Thijs et al., 2007 | -0.068 | -0.500 | 0.364 |
|  | COP velocity mediolateral component (displacement) |  | Thijs et al., 2007 | NR | | |
|  | COP maximal velocity mediolateral component (displacement) |  | Thijs et al., 2007 | -0.281 | -0.713 | 0.156 |
|  | COP anterior posterior component (displacement) |  | Thijs et al., 2007 | NR | | |
|  | COP velocity anterior posterior component (displacement) |  | Thijs et al., 2007 | NR | | |
|  | COP maximal velocity anterior posterior component (displacement) |  | Thijs et al., 2007 | NR | | |

Patellofemoral pain discontinuous (nominal) plantar variables

| Condition | Plantar loading parameter | Study ID/Ref | Experiment vs control | Risk ratio | 95% CI | |
| --- | --- | --- | --- | --- | --- | --- |
|  |  |  |  |  | Lower | Upper |
| Dynamic barefoot | Patellofemoral joint pain syndrome | Kaufman et al., 1999 | Pes Cavus vs. Normal | 0.7882 | 0.3045 | 2.0403 |
|  |  |  | Pes Cavus vs. Pes Planus | 1.4255 | 0.4662 | 4.3589 |
|  |  |  | Normal vs. Pes Cavus | 1.2687 | 0.4901 | 3.2838 |
|  |  |  | Normal vs. Pes Planus | 1.8085 | 0.6262 | 5.2233 |
|  |  |  | Pes Planus vs. Pes Cavus | 0.7015 | 0.2294 | 2.1450 |
|  |  |  | Pes Planus vs. Normal | 0.5529 | 0.1914 | 1.5970 |
| Dynamic with shoes | Patellofemoral joint pain syndrome | Kaufman et al., 1999 | Pes Cavus vs. Normal | 1.0179 | 0.3387 | 3.0584 |
|  |  |  | Pes Cavus vs. Pes Planus | 0.7308 | 0.2628 | 2.0322 |
|  |  |  | Normal vs. Pes Cavus | 0.9825 | 0.3270 | 2.9521 |
|  |  |  | Normal vs. Pes Planus | 0.7179 | 0.2582 | 1.9965 |
|  |  |  | Pes Planus vs. Pes Cavus | 1.3684 | 0.4921 | 3.8055 |
|  |  |  | Pes Planus vs. Normal | 1.3929 | 0.5009 | 3.8734 |

Achilles tendinopathy continuous plantar variables

|  | Variable | | Region definition | Study ID | Effect size (ES) | Confidence interval | |
| --- | --- | --- | --- | --- | --- | --- | --- |
|  | | |  |  |  | Lower | Upper |
| **Plantar region** | | | | | | | |
| Whole foot | | Total foot contact time |  | Van Ginckel et al., 2009 | NR | | |
|  |  | COF mediolateral Component (displacement) |  | Van Ginckel et al., 2009 | -0.553 | -1.228 | 0.137 |
|  |  | COF anterior-posterior Component (displacement) |  | Van Ginckel et al., 2009 | -0.953 | -1.636 | -0.245 |
| Medial heel  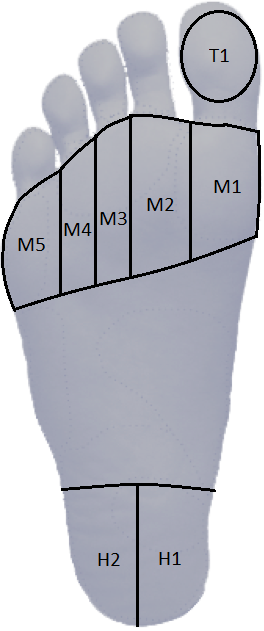  Adapted from Hesar et al. [30] | | Peak force | HM | Van Ginckel et al., 2009 | 0.322 | -0.360 | 0.996 |
|  |  | Absolute force-time integral | HM | Van Ginckel et al., 2009 | 0.161 | -0.517 | 0.836 |
|  |  | Relative force-time integral | HM | Van Ginckel et al., 2009 | 0.122 | -0.556 | 0.796 |
|  |  | Time to peak force | HM | Van Ginckel et al., 2009 | -0.716 | -1.390 | -0.020 |
|  |  | Relative Time to peak force | HM | Van Ginckel et al., 2009 | NR | | |
|  |  | Time at which region made contact | HM | Van Ginckel et al., 2009 | NR | | |
|  |  | Time at which the region ended contact | HM | Van Ginckel et al., 2009 | 0.000 | -0.676 | 0.676 |

| Lateral heel  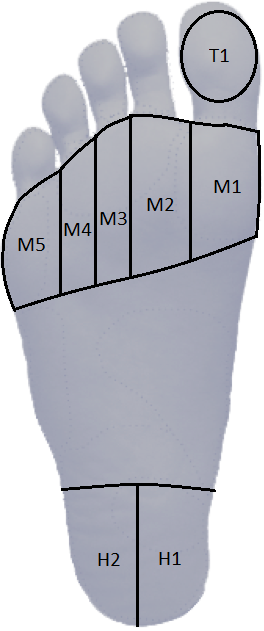  Adapted from Hesar et al. [30] | Peak force | HL | Van Ginckel et al., 2009 | 0.454 | -0.232 | 1.129 |
| --- | --- | --- | --- | --- | --- | --- |
|  | Absolute force-time integral | HL | Van Ginckel et al., 2009 | 0.121 | -0.557 | 0.796 |
|  | Relative force-time integral | HL | Van Ginckel et al., 2009 | 0.023 | -0.653 | 0.699 |
|  | Time to peak force | HL | Van Ginckel et al., 2009 | -1.083 | -1.771 | -0.368 |
|  | Relative Time to peak force | HL | Van Ginckel et al., 2009 | NR | | |
|  | Time at which region made contact | HL | Van Ginckel et al., 2009 | NR | | |
|  | Time at which the region ended contact | HL | Van Ginckel et al., 2009 | -0.500 | -1.175 | 0.187 |
| Medial forefoot (M1-M3)  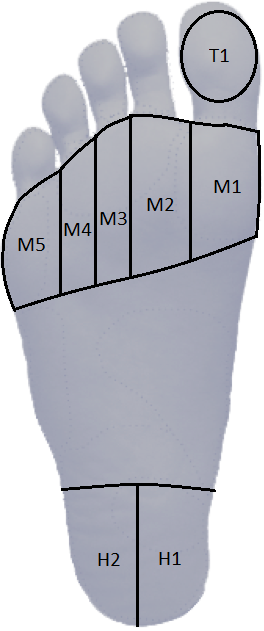  Adapted from Hesar et al. [30] | Peak force | M1 | Van Ginckel et al., 2009 | 0.209 | -0.470 | 0.883 |
|  |  | M2 |  | 0.048 | -0.628 | 0.723 |
|  |  | M3 |  | 0.183 | -0.495 | 0.858 |
|  | Absolute force-time integral | M1 | Van Ginckel et al., 2009 | 0.196 | -0.483 | 0.870 |
|  |  | M2 |  | 0.055 | -0.622 | 0.730 |
|  |  | M3 |  | 0.204 | -0.475 | 0.878 |
|  | Relative force-time integral | M1 | Van Ginckel et al., 2009 | 0.103 | -0.574 | 0.778 |
|  |  | M2 |  | -0.162 | -0.836 | 0.517 |
|  |  | M3 |  | 0.287 | -0.395 | 0.961 |
|  | Time to peak force | M1 | Van Ginckel et al., 2009 | 0.000 | -0.676 | 0.676 |
|  |  | M2 |  | 0.000 | -0.676 | 0.676 |
|  |  | M3 |  | -0.500 | -1.175 | 0.187 |
|  | Relative Time to peak force | M1 | Van Ginckel et al., 2009 | NR | | |
|  |  | M2 |  | NR | | |
|  |  | M3 |  | NR | | |
|  | Time at which region made contact | M1 | Van Ginckel et al., 2009 | 0.000 | -0.676 | 0.676 |
|  |  | M2 |  | -1.000 | -1.685 | -0.290 |
|  |  | M3 |  | 0.000 | -0.676 | 0.676 |
|  | Time at which the region ended contact | M1 | Van Ginckel et al., 2009 | -0.333 | -1.007 | 0.349 |
|  |  | M2 |  | -0.333 | -1.007 | 0.349 |
|  |  | M3 |  | -0.333 | -1.007 | 0.349 |
| Lateral forefoot (M4 and M5)  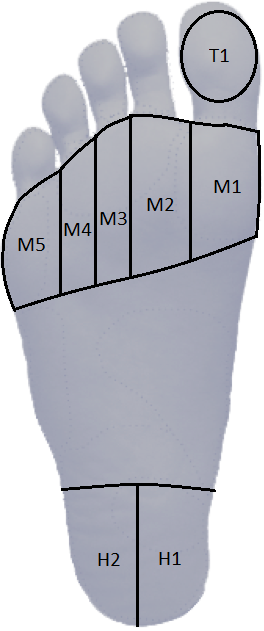  Adapted from Hesar et al. [30] | Peak force | M4 | Van Ginckel et al., 2009 | 0.404 | -0.280 | 1.078 |
|  |  | M5 |  | 0.843 | 0.142 | 1.524 |
|  | Absolute force-time integral | M4 | Van Ginckel et al., 2009 | 0.421 | -0.264 | 1.096 |
|  |  | M5 |  | 0.808 | 0.108 | 1.488 |
|  | Relative force-time integral | M4 | Van Ginckel et al., 2009 | 0.273 | -0.407 | 0.947 |
|  |  | M5 |  | 0.590 | -0.101 | 1.266 |
|  | Time to peak force | M4 | Van Ginckel et al., 2009 | 0.000 | -0.676 | 0.676 |
|  |  | M5 |  | -0.500 | -1.175 | 0.187 |
|  | Relative Time to peak force | M4 | Van Ginckel et al., 2009 | NR | | |
|  |  | M5 |  | NR | | |
|  | Time at which region made contact | M4 | Van Ginckel et al., 2009 | 0.000 | -0.676 | 0.676 |
|  |  | M5 |  | -0.530 | -1.205 | 0.158 |
|  | Time at which the region ended contact | M4 | Van Ginckel et al., 2009 | -0.333 | -1.007 | 0.349 |
|  |  | M5 |  | 0.000 | -0.676 | 0.676 |
| Hallux (T1)  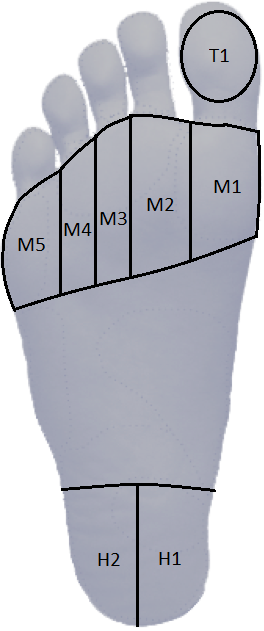  Adapted from Hesar et al. [30] | Peak Force | T1 | Van Ginckel et al., 2009 | -0.117 | -0.792 | 0.560 |
|  | Absolute force-time integral | T1 | Van Ginckel et al., 2009 | -0.199 | -0.873 | 0.480 |
|  | Relative force-time integral\ | T1 | Van Ginckel et al., 2009 | -0.146 | -0.821 | 0.532 |
|  | Time to peak force | T1 | Van Ginckel et al., 2009 | -0.316 | -0.990 | 0.366 |
|  | Relative Time to peak force | T1 | Van Ginckel et al., 2009 | NR | | |
|  | Time at which region made contact | T1 | Van Ginckel et al., 2009 | 0.416 | -0.268 | 1.091 |
|  | Time at which the region ended contact | T1 | Van Ginckel et al., 2009 | -0.250 | -0.924 | 0.430 |
| **Time specific variables during stance phase** | | | | | | |
| FFC (first foot contact) | Force ratios | Force ratio 1  [(H1 + M1 + T1) - (H2 + m4 + m5)]/sum(T1 : H2) | Van Ginckel et al., 2009 | 0.000 | -0.676 | 0.676 |
|  |  | Force ratio 2  (M1 - M5)/sum(M1:M5) |  | 0.271 | -0.410 | 0.945 |
|  | COF mediolateral Component (position |  | Van Ginckel et al., 2009 | 0.200 | -0.479 | 0.874 |
|  | COF anterior posterior component (position) |  | Van Ginckel et al., 2009 | NR | | |
| FMC (first metatarsal contact) | Time to FMC |  | Van Ginckel et al., 2009 | 0.000 | -0.676 | 0.676 |
|  | Force ratios | Force Ratio 1 [(H1 + M1 + T1) - (H2 + m4 + m5)]/sum(T1 : H2) | Van Ginckel et al., 2009 | -0.250 | -0.924 | 0.430 |
|  |  | Force ratio 2 (M1 - M5)/sum(M1:M5) |  | -0.424 | -1.098 | 0.261 |
|  | COF mediolateral component (position) |  | Van Ginckel et al., 2009 | 0.225 | -0.455 | 0.899 |
|  | COF anterior- posterior component (position) |  | Van Ginckel et al., 2009 | -0.504 | -1.179 | 0.184 |

| FFF (forefoot flat) | Time to FFF |  | Van Ginckel et al., 2009 | -0.530 | -1.205 | 0.158 |
| --- | --- | --- | --- | --- | --- | --- |
|  | Force ratios | Force ratio 1  [(H1 + M1 + T1) - (H2 + m4 + m5)]/sum(T1 : H2) | Van Ginckel et al., 2009 | -0.507 | -1.182 | 0.181 |
|  |  | Force ratio 2  (M1 - M5)/sum(M1:M5) |  | -0.884 | -1.565 | -0.180 |
|  | COF mediolateral component (position) |  | Van Ginckel et al., 2009 | 0.206 | -0.473 | 0.880 |
|  | COF anterior posterior component (position |  |  | -0.156 | -0.830 | 0.523 |
| HO (heel off) | Time to HO |  | Van Ginckel et al., 2009 | 0.000 | -0.676 | 0.676 |
|  | Force ratios | Force ratio 1  [(H1 + M1 + T1) - (H2 + m4 + m5)]/sum(T1 : H2) |  | -0.546 | -1.222 | 0.143 |
|  |  | Force ratio 2  (M1 - M5)/sum(M1:M5) |  | -0.316 | -0.990 | 0.366 |
|  | COF mediolateral component (position) |  | Van Ginckel et al., 2009 | 0.134 | -0.544 | 0.808 |
|  | COF anterior posterior component (position) |  |  | -0.544 | -1.219 | 0.145 |
| LFC (last foot contact) | Force ratios | Force Ratio 1  [(H1 + M1 + T1) - (H2 + m4 + m5)]/sum(T1 : H2) | Van Ginckel et al., 2009 | -0.271 | -0.945 | 0.410 |
|  |  | Force Ratio 2  (M1 - M5)/sum(M1:M5) |  | 0.000 | -0.676 | 0.676 |
|  | COF mediolateral component (position) |  | Van Ginckel et al., 2009 | -0.430 | -1.104 | 0.256 |
|  | COF anterior posterior component (position) |  |  | -0.948 | -1.632 | -0.241 |
| **Phase specific variables during stance phase** | | | | | | |
| ICP initial contact phase | Force ratios | Force Ratio 1  [(H1 + M1 + T1) - (H2 + m4 + m5)]/sum(T1 : H2) | Van Ginckel et al., 2009 | -0.197 | -0.871 | 0.482 |
|  |  | Force Ratio 2  (M1 - M5)/sum(M1:M5) |  | -0.472 | -1.147 | 0.214 |
|  | COF mediolateral component (displacement) |  | Van Ginckel et al., 2009 | 0.024 | -0.652 | 0.700 |
|  | COF anterior posterior component (displacement) |  |  | -0.471 | -1.146 | 0.215 |
| FFCP (forefoot contract phase) | Force ratios | Force Ratio 1  [(H1 + M1 + T1) - (H2 + m4 + m5)]/sum(T1 : H2) | Van Ginckel et al., 2009 | -0.348 | -1.022 | 0.335 |
|  |  | Force Ratio 2  (M1 - M5)/sum(M1:M5) |  | 0.091 | -0.586 | 0.766 |
|  | COF mediolateral component (displacement) |  | Van Ginckel et al., 2009 | 0.085 | -0.592 | 0.759 |
|  | COF anterior posterior component (displacement) |  |  | 0.060 | -0.617 | 0.735 |
| FFP (foot flat phase) | Force ratios | Force Ratio 1  [(H1 + M1 + T1) - (H2 + m4 + m5)]/sum(T1 : H2) | Van Ginckel et al., 2009 | 0.092 | -0.585 | 0.767 |
|  |  | Force Ratio 2  (M1 - M5)/sum(M1:M5) |  | 0.641 | -0.052 | 1.317 |
|  | COF mediolateral component (displacement) |  | Van Ginckel et al., 2009 | -0.147 | -0.821 | 0.532 |
|  | COF anterior posterior component (displacement) |  |  | -0.256 | -0.930 | 0.424 |
| FFPOP (forefoot push off phase) | Force ratios | Force Ratio 1  [(H1 + M1 + T1) - (H2 + m4 + m5)]/sum(T1 : H2) | Van Ginckel et al., 2009 | 0.341 | -0.341 | 1.015 |
|  |  | Force Ratio 2  (M1 - M5)/sum(M1:M5) |  | 0.279 | -0.401 | 0.953 |
|  | COF mediolateral component (displacement) |  | Van Ginckel et al., 2009 | -0.383 | -1.057 | 0.301 |
|  | COF anterior posterior component (displacement) |  |  | -0.751 | -1.429 | -0.053 |

Achilles tendinopathy discontinuous (nominal) plantar variables

| Condition | Plantar loading parameter | Study ID/Ref | Experiment vs control | Risk ratio | 95% CI | |
| --- | --- | --- | --- | --- | --- | --- |
|  |  |  |  |  | Lower | Upper |
| Dynamic barefoot | Achilles tendonitis | Kaufman et al., 1999 | Pes Cavus vs. Normal | 1.2632 | 0.3472 | 4.5951 |
|  |  |  | Pes Cavus vs. Pes Planus | 0.6486 | 0.2191 | 1.9200 |
|  |  |  | Normal vs. Pes Cavus | 0.7917 | 0.2176 | 2.8799 |
|  |  |  | Normal vs. Pes Planus | 0.5135 | 0.1586 | 1.6625 |
|  |  |  | Pes Planus vs. Pes Cavus | 1.5417 | 0.5208 | 4.5634 |
|  |  |  | Pes Planus vs. Normal | 1.9474 | 0.6015 | 6.3047 |
| Dynamic with shoes | Achilles tendonitis | Kaufman et al., 1999 | Pes Cavus vs. Normal | 1.2340 | 0.3877 | 3.9280 |
|  |  |  | Pes Cavus vs. Pes Planus | 1.0175 | 0.3394 | 3.0503 |
|  |  |  | Normal vs. Pes Cavus | 0.8103 | 0.2546 | 2.5793 |
|  |  |  | Normal vs. Pes Planus | 0.8246 | 0.2597 | 2.6176 |
|  |  |  | Pes Planus vs. Pes Cavus | 0.9828 | 0.3278 | 2.9460 |
|  |  |  | Pes Planus vs. Normal | 1.2128 | 0.3820 | 3.8499 |

Non specific lower limb over use injury continuous variables

| Variable | Region definition | Study ID | Effect size (ES) |  |  | Confidence interval | |
| --- | --- | --- | --- | --- | --- | --- | --- |
|  | |  |  |  | | Lower | Upper |
| Plantar regions | | | | | | | |
| Whole foot | Total contact time |  | Willems et al., 2007 | -0.031 | | -0.281 | 0.219 |
| Medial heel  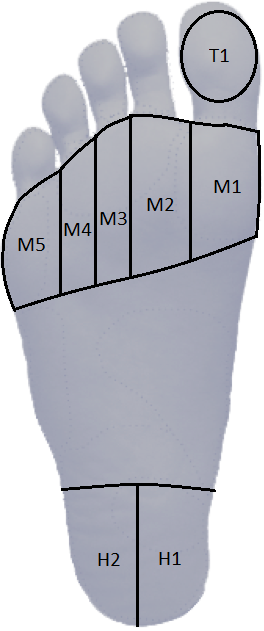  Adapted from Hesar et al. [30] | Peak force | HM | Hesar et al., 2009 | NR | | | |
|  | Absolute force-time integral | HM | Hesar et al., 2009 | NR | | | |
|  | Relative force-time integral | HM | Hesar et al., 2009 | NR | | | |
|  | Time to peak force | HM | Hesar et al., 2009 | NR | | | |
|  | Peak Pressure | H1 | Willems et al., 2006 | NR | | | |
|  |  | H1 | Willems et al., 2007 | -0.081 | | -0.331 | 0.170 |
|  | Absolute Impulses | H1 | Willems et al., 2006 | NR | | | |
|  |  | H1 | Willems et al., 2007 | -0.190 | | -0.440 | 0.062 |
|  | Time to peak pressure | H1 | Willems et al., 2007 | NR | | | |
|  | Time at which region made contact | HM | Hesar et al., 2009 | NR | | | |
|  |  | H1 | Willems et al., 2006 | NR | | | |
|  |  | H1 | Willems et al., 2007 | 0.078 | | -0.173 | 0.328 |
|  | Time at which the region ended contact | HM | Hesar et al., 2009 | NR | | | |
|  |  | H1 | Willems et al., 2006 | NR | | | |
|  |  | H1 | Willems et al., 2007 | -0.175 | | -0.426 | 0.076 |
| Lateral heel  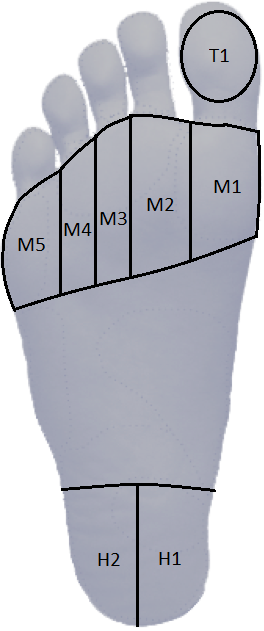  Adapted from Hesar et al. [30] | Peak force | HL | Hesar et al., 2009 | NR | | | |
|  | Absolute force-time integral | HL | Hesar et al., 2009 | NR | | | |
|  | Relative force-time integral | HL | Hesar et al., 2009 | NR | | | |
|  | Time to peak force | HL | Hesar et al., 2009 | NR | | | |
|  | Peak Pressure | H2 | Willems et al., 2006 | NR | | | |
|  |  | H2 | Willems et al., 2007 | -0.171 | | -0.421 | 0.080 |
|  | Absolute Impulses | H2 | Willems et al., 2006 | NR | | | |
|  |  | H2 | Willems et al., 2007 | -0.244 | | -0.494 | 0.008 |
|  | Time to peak pressure | H2 | Willems et al., 2007 | NR | | | |
|  | Time at which region made contact | HL | Hesar et al., 2009 | NR | | | |
|  |  | H2 | Willems et al., 2006 | 0.604 | | 0.349 | 0.857 |
|  |  | H2 | Willems et al., 2007 | 0.090 | | -0.160 | 0.341 |
|  | Time at which the region ended contact | HL | Hesar et al., 2009 | NR | | | |
|  |  | H2 | Willems et al., 2006 | NR | | | |
|  |  | H2 | Willems et al., 2007 | -0.243 | | -0.493 | 0.009 |
| Medial forefoot (M1-M3)  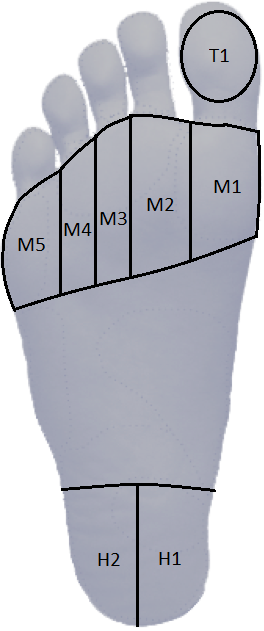  Adapted from Hesar et al. [30] | Peak force | M1 | Hesar et al., 2009 | NR | | | |
|  |  | M2 |  | NR | | | |
|  |  | M3 |  | NR | | | |
|  | Absolute force-time integral | M1 | Hesar et al., 2009 | NR | | | |
|  |  | M2 |  | NR | | | |
|  |  | M3 |  | NR | | | |
|  | Relative force-time integral | M1 | Hesar et al., 2009 | NR | | | |
|  |  | M2 |  | NR | | | |
|  |  | M3 |  | NR | | | |
|  | Time to peak force | M1 | Hesar et al., 2009 | NR | | | |
|  |  | M2 |  | NR | | | |
|  |  | M3 |  | NR | | | |
|  | Peak Pressure | M1 | Willems et al., 2006 | NR | | | |
|  |  | M2 |  | NR | | | |
|  |  | M3 |  | NR | | | |
|  |  | M1 | Willems et al., 2007 | 0.229 | | -0.022 | 0.480 |
|  |  | M2 |  | -0.060 | | -0.310 | 0.191 |
|  |  | M3 |  | -0.122 | | -0.372 | 0.129 |
|  | Absolute Impulses | M1 | Willems et al., 2006 | NR | | | |
|  |  | M2 |  | NR | | | |
|  |  | M3 |  | NR | | | |
|  |  | M1 | Willems et al., 2007 | 0.158 | | -0.093 | 0.408 |
|  |  | M2 |  | -0.053 | | -0.303 | 0.198 |
|  |  | M3 |  | -0.088 | | -0.339 | 0.162 |
|  | Time to peak pressure | M1 | Willems et al., 2007 | NR | | | |
|  |  | M2 |  | NR | | | |
|  |  | M3 |  | NR | | | |
|  | Time at which region made contact | M1 | Hesar et al., 2009 | NR | | | |
|  |  | M2 |  | NR | | | |
|  |  | M3 |  | NR | | | |
|  |  | M1 | Willems et al., 2006 | NR | | | |
|  |  | M2 |  | NR | | | |
|  |  | M3 |  | NR | | | |
|  |  | M1 | Willems et al., 2007 | -0.196 | | -0.446 | 0.056 |
|  |  | M2 |  | -0.101 | | -0.351 | 0.150 |
|  |  | M3 |  | -0.062 | | -0.312 | 0.189 |
|  | Time at which the region ended contact | M1 | Hesar et al., 2009 | NR | | | |
|  |  | M2 |  | NR | | | |
|  |  | M3 |  | NR | | | |
|  |  | M1 | Willems et al., 2006 | NR | | | |
|  |  | M2 |  | 0.430 | | 0.177 | 0.681 |
|  |  | M3 |  | 0.369 | | 0.117 | 0.620 |
|  |  | M1 | Willems et al., 2007 | -0.180 | | -0.431 | 0.071 |
|  |  | M2 |  | -0.032 | | -0.282 | 0.219 |
|  |  | M3 |  | 0.046 | | -0.204 | 0.296 |
| Lateral forefoot (M4 and M5)  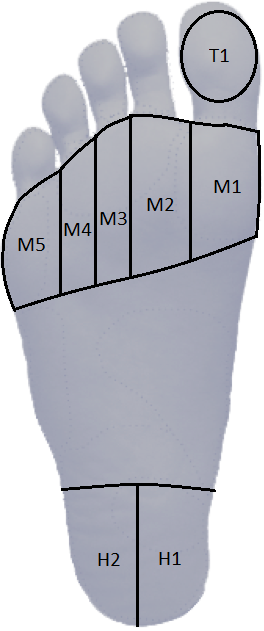  Adapted from Hesar et al. [30] | Peak force | M4 | Hesar et al., 2009 | NR | | | |
|  |  | M5 |  | 0.524 | | 0.093 | 0.949 |
|  | Absolute force-time integral | M4 | Hesar et al., 2009 | NR | | | |
|  |  | M5 |  | 0.573 | | 0.141 | 0.999 |
|  | Relative force-time integral | M4 | Hesar et al., 2009 | NR | | | |
|  |  | M5 |  | NR | | | |
|  | Time to peak force | M4 | Hesar et al., 2009 | NR | | | |
|  |  | M5 |  | NR | | | |
|  | Peak pressure | M4 | Willems et al., 2006 | NR | | | |
|  |  | M5 |  | -0.444 | | -0.696 | -0.191 |
|  |  | M4 | Willems et al., 2007 | -0.179 | | -0.429 | 0.073 |
|  |  | M5 |  | -0.225 | | -0.476 | 0.026 |
|  | Absolute Impulses | M4 | Willems et al., 2006 | NR | | | |
|  |  | M5 |  | -0.419 | | -0.670 | -0.166 |
|  |  | M4 | Willems et al., 2007 | -0.199 | | -0.449 | 0.053 |
|  |  | M5 |  | -0.306 | | -0.557 | -0.054 |
|  | Time to peak pressure | M4 | Willems et al., 2007 | NR | | | |
|  |  | M5 |  | NR | | | |
|  | Time at which region made contact | M4 | Hesar et al., 2009 | NR | | | |
|  |  | M5 |  | NR | | | |
|  |  | M4 | Willems et al., 2006 | NR | | | |
|  |  | M5 |  | 0.321 | | 0.069 | 0.571 |
|  |  | M4 | Willems et al., 2007 | 0.055 | | -0.196 | 0.305 |
|  |  | M5 |  | 0.114 | | -0.137 | 0.364 |
|  | Time at which the region ended contact | M4 | Hesar et al., 2009 | NR | | | |
|  |  | M5 |  | NR | | | |
|  |  | M4 | Willems et al., 2006 | NR | | | |
|  |  | M5 |  | NR | | | |
|  |  | M4 | Willems et al., 2007 | -0.087 | | -0.338 | 0.163 |
|  |  | M5 |  | -0.189 | | -0.440 | 0.062 |
| Hallux (T1)  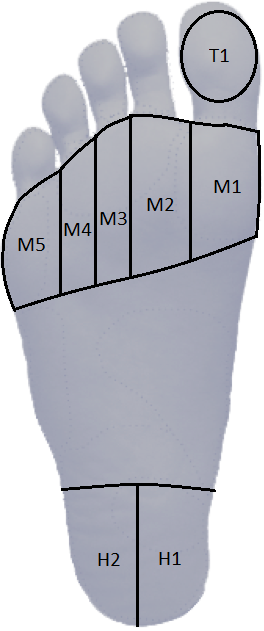  Adapted from Hesar et al. [30] | Peak Force | T1 | Hesar et al., 2009 | NR | | | |
|  | Absolute force-time integral | T1 | Hesar et al., 2009 | NR | | | |
|  | Relative force-time integral | T1 | Hesar et al., 2009 | NR | | | |
|  | Time to peak force | T1 | Hesar et al., 2009 | NR | | | |
|  | Peak pressure | T1 | Willems et al., 2006 | NR | | | |
|  |  | T1 | Willems et al., 2007 | 0.195 | | -0.056 | 0.446 |
|  | Absolute Impulses | T1 | Willems et al., 2006 | NR | | | |
|  |  | T1 | Willems et al., 2007 | 0.115 | | -0.136 | 0.365 |
|  | Time to peak Pressure | T1 | Willems et al., 2007 | NR | | | |
|  | Time at which region made contact | T1 | Hesar et al., 2009 | NR | | | |
|  |  | T1 | Willems et al., 2006 | NR | | | |
|  |  | T1 | Willems et al., 2007 | -0.053 | | -0.303 | 0.198 |
|  | Time at which the region ended contact | T1 | Hesar et al., 2009 | NR | | | |
|  |  | T1 | Willems et al., 2006 | NR | | | |
|  |  | T1 | Willems et al., 2007 | 0.090 | | -0.160 | 0.341 |

| Time specific variables during stance phase | | | | | | |
| --- | --- | --- | --- | --- | --- | --- |
| FFC (first foot contact) | Force ratios | Force ratio 1  [(H1 + M1 + T1) - (H2 + m4 + m5)]/sum(T1 : H2) | Hesar et al., 2009 | NR | | |
|  |  | Force ratio 2  [(H1 + M1 + M2) - (H2 + m4 + m5)]/sum(T1 : H2) |  | NR | | |
|  |  | Force ratio 3  [(H1 + M1) - (H2 + M4 + m5)/sum(T1 : H2) |  | NR | | |
|  |  | Force ratio 4  [(M1 + M2) - (M4 + M5)]/sum(M1 : M5)] |  | NR | | |
|  |  | Force ratio 5  [(M1 + M2) - (M3 + M4 + M5)]/sum(M1 : M5)] |  | NR | | |
|  |  | Force ratio 6  (M1 - M5)/sum(M1 : M5) |  | NR | | |
|  |  | Force ratio 7  (M1 + M2)/(M1 + M2 + M4 + M5) |  | NR | | |
|  |  | Force ratio 8 (M1 + M2 + H1)/(M4 + M5 + H2) |  | NR | | |
|  |  | Force ratio 9 [(M1 + M2) - (M4 + M5)]/(M1 + M2 + M4 + M5) |  | NR | | |
|  | COF mediolateral component (position) |  | Hesar et al., 2009 | NR | | |
|  | COF velocity mediolateral component |  | Hesar et al., 2009 | NR | | |
|  | COF anterior posterior component (position) |  | Hesar et al., 2009 | NR | | |
|  | COF velocity anterior- posterior component |  | Hesar et al., 2009 | NR | | |
|  | Pressure ratios | Mediolateral pressure ratio  [(HM + M1 + M2) - (HL + m4 + m5)]/sum(T1 : H2) | Willems et al., 2006 | NR | | |
|  |  | Mediolateral pressure ratio  [(HM + M1 + M2) - (HL + m4 + m5)]/100*sum(T1 : H2) | Willems et al., 2007 | NR | | |
|  | COP mediolateral component (position) | % of shoe width and length | Willems et al., 2006 | NR | | |
|  |  | % of shoe width and length | Willems et al., 2007 | -0.474 | -0.726 | -0.221 |
|  | COP Anterior posterior component (position) | % of shoe width and length | Willems et al., 2006 | 0.306 | 0.055 | 0.557 |
|  |  | % of shoe width and length | Willems et al., 2007 | -0.097 | -0.347 | 0.154 |

| FMC (first metatarsal contact) | Time to FMC |  | Willems et al., 2007 | 0.049 | -0.202 | 0.299 |
| --- | --- | --- | --- | --- | --- | --- |
|  | Force ratios | Force ratio 1  [(H1 + M1 + T1) - (H2 + m4 + m5)]/sum(T1 : H2) | Hesar et al., 2009 | -0.014 | -0.438 | 0.409 |
|  |  | Force ratio 2  [(H1 + M1 + M2) - (H2 + m4 + m5)]/sum(T1 : H2) |  | -0.548 | -0.973 | -0.116 |
|  |  | Force ratio 3  [(H1 + M1) - (H2 + M4 + m5)/sum(T1 : H2) |  | -0.565 | -0.990 | -0.133 |
|  |  | Force ratio 4  [(M1 + M2) - (M4 + M5)]/sum(M1 : M5)] |  | NR | | |
|  |  | Force ratio 5 [(M1 + M2) - (M3 + M4 + M5)]/sum(M1 : M5)] |  | NR | | |
|  |  | Force ratio 6 (M1 - M5)/sum(M1 : M5) |  | NR | | |
|  |  | Force ratio 7 (M1 + M2)/(M1 + M2 + M4 + M5) |  | NR | | |
|  |  | Force ratio 8 (M1 + M2 + H1)/(M4 + M5 + H2) |  | -0.592 | -1.018 | -0.159 |
|  |  | Force ratio 9 [(M1 + M2) - (M4 + M5)]/(M1 + M2 + M4 + M5) |  | NR | | |

|  | COF mediolateral component (position) |  | Hesar et al., 2009 | NR | | |
| --- | --- | --- | --- | --- | --- | --- |
|  | COF velocity mediolateral component |  | Hesar et al., 2009 | NR | | |
|  | COF anterior- posterior component (position) |  | Hesar et al., 2009 | NR | | |
|  | COF velocity anterior posterior component |  | Hesar et al., 2009 | NR | | |
|  | Pressure ratios | Mediolateral pressure ratio  [(HM + M1 + M2) - (HL + m4 + m5)]/sum(T1 : H2) | Willems et al., 2006 | NR | | |
|  |  | Mediolateral pressure ratio  [(HM + M1 + M2) - (HL + m4 + m5)]/  100*sum(T1 : H2) | Willems et al., 2007 | 0.185 | -0.067 | 0.435 |
|  | COP mediolateral component (position) | % of shoe width and length | Willems et al., 2006 | NR | | |
|  |  | % of shoe width and length | Willems et al., 2007 | -0.197 | -0.447 | 0.054 |
|  | COP anterior- posterior component (position) | % of shoe width and length | Willems et al., 2006 | NR | | |
|  |  | % of shoe width and length | Willems et al., 2007 | 0.066 | -0.184 | 0.317 |

| FFF (forefoot flat) | Time to FFF |  | Willems et al., 2007 | -0.194 | -0.444 | 0.058 |
| --- | --- | --- | --- | --- | --- | --- |
|  | Force ratios | Force ratio 1 [(H1 + M1 + T1) - (H2 + m4 + m5)]/sum(T1 : H2) | Hesar et al., 2009 | -0.847 | -1.277 | -0.406 |
|  |  | Force ratio 2  [(H1 + M1 + M2) - (H2 + m4 + m5)]/sum(T1 : H2) |  | NR | | |
|  |  | Force ratio 3  [(H1 + M1) - (H2 + M4 + m5)/sum(T1 : H2) foot |  | -0.471 | -0.895 | -0.041 |
|  |  | Force ratio 4  [(M1 + M2) - (M4 + M5)]/sum(M1 : M5)] |  | NR | | |
|  |  | Force ratio 5  [(M1 + M2) - (M3 + M4 + M5)]/sum(M1 : M5)] |  | NR | | |
|  |  | Force ratio 6  (M1 - M5)/sum(M1 : M5) |  | NR | | |
|  |  | Force ratio 7  (M1 + M2)/(M1 + M2 + M4 + M5) |  | NR | | |
|  |  | Force ratio 8  (M1 + M2 + H1)/(M4 + M5 + H2) |  | NR | | |
|  |  | Force ratio 9  [(M1 + M2) - (M4 + M5)]/(M1 + M2 + M4 + M5) |  | NR | | |
|  | COF mediolateral component (position) |  | Hesar et al., 2009 | NR | | |
|  | COF velocity mediolateral component |  | Hesar et al., 2009 | -0.640 | -1.066 | -0.206 |
|  | COF anterior posterior component (position) |  | Hesar et al., 2009 | 0.612 | 0.178 | 1.038 |
|  | COF velocity anterior posterior component |  | Hesar et al., 2009 | -0.455 | -0.879 | -0.025 |
|  | Pressure ratios | Mediolateral pressure ratio  [(HM + M1 + M2) - (HL + m4 + m5)]/ 100sum(T1 : H2) | Willems et al., 2006 | 0.472 | 0.219 | 0.724 |
|  |  | Mediolateral pressure ratio  [(HM + M1 + M2) - (HL + m4 + m5)]/100*sum(T1 : H2) | Willems et al., 2007 | 0.339 | 0.086 | 0.589 |
|  | COP mediolateral component (position) | % of shoe width and length | Willems et al., 2006 | 0.380 | 0.127 | 0.631 |
|  |  | % of shoe width and length | Willems et al., 2007 | 0.141 | -0.110 | 0.392 |
|  | COP anterior posterior component (position) | % of shoe width and length | Willems et al., 2006 | NR | | |
|  |  | % of shoe width and length | Willems et al., 2007 | -0.205 | -0.456 | 0.046 |

| HO (heel off) | Time to HO |  | Willems et al., 2007 | -0.097 | -0.347 | 0.154 |
| --- | --- | --- | --- | --- | --- | --- |
|  | Force ratios | Force ratio 1  [(H1 + M1 + T1) - (H2 + m4 + m5)]/sum(T1 : H2) | Hesar et al., 2009 | NR | | |
|  |  | Force ratio 2  [(H1 + M1 + M2) - (H2 + m4 + m5)]/sum(T1 : H2) |  | NR | | |
|  |  | Force ratio 3  [(H1 + M1) - (H2 + M4 + m5)/sum(T1 : H2) |  | NR | | |
|  |  | Force ratio 4  [(M1 + M2) - (M4 + M5)]/sum(M1 : M5)] |  | NR | | |
|  |  | Force ratio 5  [(M1 + M2) - (M3 + M4 + M5)]/sum(M1 : M5)] |  | NR | | |
|  |  | Force ratio 6  (M1 - M5)/sum(M1 : M5) |  | NR | | |
|  |  | Force ratio 7  (M1 + M2)/(M1 + M2 + M4 + M5) |  | NR | | |
|  |  | Force ratio 8  (M1 + M2 + H1)/(M4 + M5 + H2) |  | NR | | |
|  |  | Force ratio 9  [(M1 + M2) - (M4 + M5)]/(M1 + M2 + M4 + M5) |  | NR | | |
|  | COF mediolateral component (position) |  | Hesar et al., 2009 | -0.704 | -1.131 | -0.268 |
|  | COF velocity mediolateral component |  | Hesar et al., 2009 | NR | | |
|  | COF anterior posterior component (position) |  | Hesar et al., 2009 | NR | | |
|  | COF velocity anterior posterior component |  | Hesar et al., 2009 | NR | | |
|  | Pressure ratios | Mediolateral Pressure ratio  [(HM + M1 + M2) - (HL + m4 + m5)]/ 100*sum(T1 : H2) | Willems et al., 2006 | 0.326 | 0.074 | 0.577 |
|  |  | Mediolateral Pressure ratio  [(HM + M1 + M2) - (HL + m4 + m5)]/100*sum(T1 : H2) | Willems et al., 2007 | 0.333 | 0.081 | 0.584 |
|  | COP mediolateral component (position) | % of shoe width and length | Willems et al., 2006 | NR | | |
|  |  | % of shoe width and length | Willems et al., 2007 | 0.228 | -0.023 | 0.479 |
|  | COP anterior posterior Component (position) | % of shoe width and length | Willems et al., 2006 | NR | | |
|  |  | % of shoe width and length | Willems et al., 2007 | -0.220 | -0.470 | 0.032 |

| LFC (last foot contact) | Force ratios | Force Ratio 1  [(H1 + M1 + T1) - (H2 + m4 + m5)]/sum(T1 : H2) | Hesar et al., 2009 | NR | | |
| --- | --- | --- | --- | --- | --- | --- |
|  |  | Force Ratio 2  [(H1 + M1 + M2) - (H2 + m4 + m5)]/sum(T1 : H2) |  | NR | | |
|  |  | Force Ratio 3  [(H1 + M1) - (H2 + M4 + m5)/sum(T1 : H2) |  | NR | | |
|  |  | Force Ratio 4  [(M1 + M2) - (M4 + M5)]/sum(M1 : M5)] |  | NR | | |
|  |  | Force Ratio 5  [(M1 + M2) - (M3 + M4 + M5)]/sum(M1 : M5)] |  | NR | | |
|  |  | Force Ratio 6  (M1 - M5)/sum(M1 : M5) |  | NR | | |
|  |  | Force Ratio 7  (M1 + M2)/(M1 + M2 + M4 + M5) |  | NR | | |
|  |  | Force Ratio 8 "  (M1 + M2 + H1)/(M4 + M5 + H2) Medial foot / Lateral foot |  | NR | | |
|  |  | Force Ratio 9  [(M1 + M2) - (M4 + M5)]/(M1 + M2 + M4 + M5) |  | NR | | |
|  | COF mediolateral component (position) |  | Hesar et al., 2009 | NR | | |
|  | COF velocity mediolateral component |  | Hesar et al., 2009 | NR | | |
|  | COF anterior posterior component (position) |  | Hesar et al., 2009 | NR | | |
|  | COF velocity anterior posterior component |  | Hesar et al., 2009 | NR | | |
|  | Pressure ratios | Mediolateral Pressure ratio  [(HM + M1 + M2) - (HL + m4 + m5)]/ 100sum(T1 : H2) | Willems et al., 2006 | NR | | |
|  |  | Mediolateral Pressure ratio  [(HM + M1 + M2) - (HL + m4 + m5)]/100*sum(T1 : H2) | Willems et al., 2007 | NR | | |
|  | COP mediolateral component (position) | % of shoe width and length | Willems et al., 2006 | -0.809 | -1.064 | -0.551 |
|  |  | % of shoe width and length | Willems et al., 2007 | -0.162 | -0.412 | 0.089 |
|  | COP anterior posterior component (position) | % of shoe width and length | Willems et al., 2006 | -0.533 | -0.785 | -0.279 |
|  |  | % of shoe width and length | Willems et al., 2007 | -0.031 | -0.282 | 0.219 |

| ICP initial contact phase | Force ratios | Force ratio 1  [(H1 + M1 + T1) - (H2 + m4 + m5)]/sum(T1 : H2) | Hesar et al., 2009 | NR | | |
| --- | --- | --- | --- | --- | --- | --- |
|  |  | Force ratio 2  [(H1 + M1 + M2) - (H2 + m4 + m5)]/sum(T1 : H2) |  | NR | | |
|  |  | Force ratio 3  [(H1 + M1) - (H2 + M4 + m5)/sum(T1 : H2) |  | NR | | |
|  |  | Force ratio 4  [(M1 + M2) - (M4 + M5)]/sum(M1 : M5)] |  | NR | | |
|  |  | Force ratio 5  [(M1 + M2) - (M3 + M4 + M5)]/sum(M1 : M5)] |  | NR | | |
|  |  | Force ratio 6  (M1 - M5)/sum(M1 : M5) |  | NR | | |
|  |  | Force ratio 7  (M1 + M2)/(M1 + M2 + M4 + M5) |  | NR | | |
|  |  | Force ratio 8 (M1 + M2 + H1)/(M4 + M5 + H2) |  | -0.430 | -0.854 | -0.001 |
|  |  | Force ratio 9 [(M1 + M2) - (M4 + M5)]/(M1 + M2 + M4 + M5) |  | NR | | |
|  | COF mediolateral component (displacement) |  | Hesar et al., 2009 | NR | | |
|  | COF velocity mediolateral component (displacement) |  | Hesar et al., 2009 | NR | | |
|  | COF anterior posterior component (displacement) |  | Hesar et al., 2009 | NR | | |
|  | COF velocity anterior posterior Component (displacement) |  | Hesar et al., 2009 | NR | | |
|  | Pressure ratios | Mediolateral pressure ratio  [(HM + M1 + M2) - (HL + m4 + m5)]/sum(T1 : H2) | Willems et al., 2006 | NR | | |
|  |  | Mediolateral pressure ratio  [(HM + M1 + M2) - (HL + m4 + m5)]/100*sum(T1 : H2) | Willems et al., 2007 | 0.568 | 0.313 | 0.820 |
|  | COP mediolateral component (displacement) | % of shoe width and length | Willems et al., 2006 | NR | | |
|  |  | % of shoe width and length | Willems et al., 2007 | 0.611 | 0.356 | 0.863 |
|  | COP anterior posterior component (displacement) | % of shoe width and length | Willems et al., 2006 | NR | | |
|  |  | % of shoe width and length | Willems et al., 2007 | 0.111 | -0.140 | 0.361 |

| FFCP (forefoot contract phase) | Force ratios | Force ratio 1  [(H1 + M1 + T1) - (H2 + m4 + m5)]/sum(T1 : H2) | Hesar et al., 2009 | NR | | |
| --- | --- | --- | --- | --- | --- | --- |
|  |  | Force ratio 2  [(H1 + M1 + M2) - (H2 + m4 + m5)]/sum(T1 : H2) |  | NR | | |
|  |  | Force ratio 3  [(H1 + M1) - (H2 + M4 + m5)/sum(T1 : H2) |  | NR | | |
|  |  | Force ratio 4  [(M1 + M2) - (M4 + M5)]/sum(M1 : M5)] |  | NR | | |
|  |  | Force ratio 5  [(M1 + M2) - (M3 + M4 + M5)]/sum(M1 : M5)] |  | NR | | |
|  |  | Force ratio 6  (M1 - M5)/sum(M1 : M5) |  | NR | | |
|  |  | Force ratio 7  (M1 + M2)/(M1 + M2 + M4 + M5) |  | NR | | |
|  |  | Force ratio 8 (M1 + M2 + H1)/(M4 + M5 + H2) |  | NR | | |
|  |  | Force ratio 9 [(M1 + M2) - (M4 + M5)]/(M1 + M2 + M4 + M5) |  | NR | | |
|  | COF mediolateral component (displacement) |  | Hesar et al., 2009 | -0.842 | -1.272 | -0.402 |
|  | COF velocity mediolateral component (displacement) |  | Hesar et al., 2009 | NR | | |
|  | COF anterior posterior component (displacement) |  | Hesar et al., 2009 | NR | | |
|  | COF velocity anterior posterior component (displacement) |  | Hesar et al., 2009 | NR | | |
|  | Pressure ratios | Mediolateral pressure ratio  [(HM + M1 + M2) - (HL + m4 + m5)]/sum(T1 : H2) | Willems et al., 2006 | 0.540 | 0.285 | 0.792 |
|  |  | Mediolateral pressure ratio  [(HM + M1 + M2) - (HL + m4 + m5)]/100*sum(T1 : H2) | Willems et al., 2007 | 0.104 | -0.146 | 0.355 |
|  | COP mediolateral component (displacement) | % of shoe width and length | Willems et al., 2006 | 0.582 | 0.327 | 0.834 |
|  |  | % of shoe width and length | Willems et al., 2007 | 0.305 | 0.053 | 0.555 |
|  | COP anterior posterior component (displacement) | % of shoe width and length | Willems et al., 2006 | NR | | |
|  |  | % of shoe width and length | Willems et al., 2007 | -0.227 | -0.477 | 0.025 |

| FFP (foot flat phase) | Force ratios | Force ratio 1  [(H1 + M1 + T1) - (H2 + m4 + m5)]/sum(T1 : H2) | Hesar et al., 2009 | NR | | |
| --- | --- | --- | --- | --- | --- | --- |
|  |  | Force ratio 2  [(H1 + M1 + M2) - (H2 + m4 + m5)]/sum(T1 : H2) |  | NR | | |
|  |  | Force ratio 3  [(H1 + M1) - (H2 + M4 + m5)/sum(T1 : H2) |  | NR | | |
|  |  | Force ratio 4  [(M1 + M2) - (M4 + M5)]/sum(M1 : M5)] |  | NR | | |
|  |  | Force ratio 5  [(M1 + M2) - (M3 + M4 + M5)]/sum(M1 : M5)] |  | NR | | |
|  |  | Force ratio 6  (M1 - M5)/sum(M1 : M5) |  | NR | | |
|  |  | Force ratio 7  (M1 + M2)/(M1 + M2 + M4 + M5) |  | NR | | |
|  |  | Force ratio 8 (M1 + M2 + H1)/(M4 + M5 + H2) |  | NR | | |
|  |  | Force ratio 9 [(M1 + M2) - (M4 + M5)]/(M1 + M2 + M4 + M5) |  | NR | | |
|  | COF mediolateral component (displacement) |  | Hesar et al., 2009 | -0.607 | -1.033 | -0.174 |
|  | COF velocity mediolateral component (displacement) |  | Hesar et al., 2009 | NR | | |
|  | COF anterior posterior component (displacement) |  | Hesar et al., 2009 | NR | | |
|  | COF velocity anterior posterior component (displacement) |  | Hesar et al., 2009 | NR | | |
|  | Pressure ratios | Mediolateral pressure ratio  [(HM + M1 + M2) - (HL + m4 + m5)]/sum(T1 : H2) | Willems et al., 2006 | NR | | |
|  |  | Mediolateral pressure ratio  [(HM + M1 + M2) - (HL + m4 + m5)]/100*sum(T1 : H2) | Willems et al., 2007 | -0.084 | -0.334 | 0.167 |
|  | COP mediolateral component (displacement) | % of shoe width and length | Willems et al., 2006 | NR | | |
|  |  | % of shoe width and length | Willems et al., 2007 | 0.153 | -0.098 | 0.403 |
|  | COP anterior posterior component (displacement) | % of shoe width and length | Willems et al., 2006 | NR | | |
|  |  | % of shoe width and length | Willems et al., 2007 | 0.026 | -0.225 | 0.276 |

| FFPOP (forefoot push off phase) | Force ratios | Force ratio 1  [(H1 + M1 + T1) - (H2 + m4 + m5)]/sum(T1 : H2) | Hesar et al., 2009 | NR | | |
| --- | --- | --- | --- | --- | --- | --- |
|  |  | Force ratio 2  [(H1 + M1 + M2) - (H2 + m4 + m5)]/sum(T1 : H2) |  | NR | | |
|  |  | Force ratio 3  [(H1 + M1) - (H2 + M4 + m5)/sum(T1 : H2) |  | NR | | |
|  |  | Force ratio 4  [(M1 + M2) - (M4 + M5)]/sum(M1 : M5)] |  | NR | | |
|  |  | Force ratio 5  [(M1 + M2) - (M3 + M4 + M5)]/sum(M1 : M5)] |  | NR | | |
|  |  | Force ratio 6  (M1 - M5)/sum(M1 : M5) |  | NR | | |
|  |  | Force ratio 7  (M1 + M2)/(M1 + M2 + M4 + M5) |  | NR | | |
|  |  | Force ratio 8 (M1 + M2 + H1)/(M4 + M5 + H2) |  | NR | | |
|  |  | Force ratio 9 [(M1 + M2) - (M4 + M5)]/(M1 + M2 + M4 + M5) |  | NR | | |
|  | COF mediolateral component (displacement) |  | Hesar et al., 2009 | 0.519 | 0.088 | 0.944 |
|  | COF velocity mediolateral component (displacement) |  | Hesar et al., 2009 | NR | | |
|  | COF anterior posterior component (displacement) |  | Hesar et al., 2009 | NR | | |
|  | COF velocity anterior posterior component (displacement) |  | Hesar et al., 2009 | NR | | |
|  | Pressure ratios | Mediolateral pressure ratio  [(HM + M1 + M2) - (HL + m4 + m5)]/sum(T1 : H2) | Willems et al., 2006 | NR | | |
|  |  | Mediolateral pressure ratio  [(HM + M1 + M2) - (HL + m4 + m5)]/100*sum(T1 : H2) | Willems et al., 2007 | -0.346 | -0.597 | -0.094 |
|  | COP mediolateral component (displacement) | % of shoe width and length | Willems et al., 2006 | -0.837 | -1.092 | -0.578 |
|  |  | % of shoe width and length | Willems et al., 2007 | -0.369 | -0.620 | -0.117 |
|  | COP anterior posterior component (displacement) | % of shoe width and length | Willems et al., 2006 | NR | | |
|  |  | % of shoe width and length | Willems et al., 2007 | 0.307 | 0.055 | 0.558 |

Lower limb stress fractures discontinuous (nominal) plantar variables

| Condition | Plantar loading parameter | Study ID/Ref | Experiment vs control | Risk ratio | 95% CI | |
| --- | --- | --- | --- | --- | --- | --- |
|  |  |  |  |  | Lower | Upper |
| Dynamic barefoot | Stress fractures | Kaufman et al., 1999 | Pes Cavus vs. Normal | 1.7000 | 0.5900 | 4.8985 |
|  |  |  | Pes Cavus vs. Pes Planus | 0.7798 | 0.3428 | 1.7740 |
|  |  |  | Normal vs. Pes Cavus | 0.5882 | 0.2041 | 1.6950 |
|  |  |  | Normal vs. Pes Planus | 0.4587 | 0.1675 | 1.2565 |
|  |  |  | Pes Planus vs. Pes Cavus | 1.2824 | 0.5637 | 2.9172 |
|  |  |  | Pes Planus vs. Normal | 2.1800 | 0.7959 | 5.9713 |
| Dynamic with shoes | Stress fractures | Kaufman et al., 1999 | Pes Cavus vs. Normal | 1.8298 | 0.6339 | 5.2816 |
|  |  |  | Pes Cavus vs. Pes Planus | 0.7478 | 0.3292 | 1.6986 |
|  |  |  | Normal vs. Pes Cavus | 0.5465 | 0.1893 | 1.5775 |
|  |  |  | Normal vs. Pes Planus | 0.4087 | 0.1489 | 1.1215 |
|  |  |  | Pes Planus vs. Pes Cavus | 1.3372 | 0.5887 | 3.0374 |
|  |  |  | Pes Planus vs. Normal | 2.4468 | 0.8916 | 6.7146 |

Iliotibial band syndrome discontinuous (nominal) plantar variables

| Condition | Plantar loading parameter | Study ID/Ref | Experiment vs control | Risk ratio | 95% CI | |
| --- | --- | --- | --- | --- | --- | --- |
|  |  |  |  |  | Lower | Upper |
| Dynamic barefoot | Iliotibial band syndrome | Kaufman et al., 1999 | Pes Cavus vs. Normal | 1.2179 | 0.4997 | 2.9685 |
|  |  |  | Pes Cavus vs. Pes Planus | 0.9406 | 0.4169 | 2.1224 |
|  |  |  | Normal vs. Pes Cavus | 0.8211 | 0.3369 | 2.0012 |
|  |  |  | Normal vs. Pes Planus | 0.7723 | 0.3232 | 1.8454 |
|  |  |  | Pes Planus vs. Pes Cavus | 1.0632 | 0.4712 | 2.3989 |
|  |  |  | Pes Planus vs. Normal | 1.2949 | 0.5419 | 3.0942 |
| Dynamic with shoes | Iliotibial band syndrome | Kaufman et al., 1999 | Pes Cavus vs. Normal | 1.4615 | 0.5766 | 3.7044 |
|  |  |  | Pes Cavus vs. Pes Planus | 0.8261 | 0.3729 | 1.8302 |
|  |  |  | Normal vs. Pes Cavus | 0.6842 | 0.2700 | 1.7342 |
|  |  |  | Normal vs. Pes Planus | 0.5652 | 0.2310 | 1.3830 |
|  |  |  | Pes Planus vs. Pes Cavus | 1.2105 | 0.5464 | 2.6819 |
|  |  |  | Pes Planus vs. Normal | 1.7692 | 0.7231 | 4.3291 |
