# Supplementary material for: Dynamic foot function as a risk factor for lower limb overuse injury: a systematic review
Source: J Foot Ankle Res. 2014 Dec 19;7:53. doi: 10.1186/s13047-014-0053-6 (PMC4296532; doi:10.1186/s13047-014-0053-6)
Supplement: Additional file 5: — Presentation of kinematic and kinetic variables across the 12 studies. [file 13047_2014_53_MOESM5_ESM.docx]

**Additional file 5 – kinematic and kinetic analysis**

Non specific lower limb over use injury’ continuous variables

| **Kinematic variable** | **Study ID** | **Effect size (ES)** |  | **Confidence interval** | | **Statistical significance reported in paper** |
| --- | --- | --- | --- | --- | --- | --- |
|  | |  |  | **Lower** | **Upper** |  |
| **Rearfoot motion** | **Maximal eversion position** | Willems et al., 2006 | **0.373** | **0.121** | **0.624** | p=0.034 |
|  | **Eversion excursion** |  | **0.361** | **0.108** | **0.612** | p=0.032 |
|  | **Mean eversion velocity** |  | **0.368** | **0.116** | **0.619** | p=0.034 |
|  | **Maximal eversion velocity** |  | **0.389** | **0.137** | **0.641** | p=0.031 |
|  | **Mean inversion velocity** |  | **0.435** | **0.182** | **0.686** | p=0.029 |
|  | **Initial position (°)** | Willems et al., 2007 | -0.180 | -0.431 | 0.071 |  |
|  | **Maximal position (°)** |  | 0.118 | -0.132 | 0.369 |  |
|  | **Eversion excursion (°)** |  | **0.313** | **0.061** | **0.564** | NR |
|  | **Push-off position (°)** |  | -0.154 | -0.404 | 0.097 |  |
|  | **Maxmimal eversion velocity (° s-1)** |  | **0.285** | **0.034** | **0.536** | NR |
|  | **Mean eversion velocity (° s-1)** |  | 0.101 | -0.150 | 0.351 |  |
|  | **Timing of maximal eversion (% of stance phase)** |  | **0.392** | **0.140** | **0.644** | p<0.05 |
|  | **Maximal reinversion velocity (° s-1)** |  | **0.408** | **0.155** | **0.659** | p<0.05 |
|  | **Mean reinversion velocity (° s-1)** |  | **0.307** | **0.055** | **0.558** | p<0.05 |
| **Forefoot motion** | **Abduction excursion** | Willems et al., 2006 | **0.356** | **0.103** | **0.607** | p=0.026 |
|  | **Maximal abduction velocity** |  | **0.622** | **0.367** | **0.875** | p=0.001 |
|  | **Initial position (°)** | Willems et al., 2007 | -0.076 | -0.326 | 0.175 |  |
|  | **Maximal position (°)** |  | -0.003 | -0.253 | 0.248 |  |
|  | **Abduction excursion (°)** |  | **0.313** | **0.061** | **0.563** | p<0.05 |
|  | **Push-off position (°)** |  | 0.014 | -0.237 | 0.264 |  |
|  | **Maxmimal abduction velocity (° s-1)** |  | 0.230 | -0.021 | 0.481 |  |
|  | **Mean abduction velocity (° s-1)** |  | -0.055 | -0.306 | 0.195 |  |
|  | **Timing of maximal abduction (% of stance phase)** |  | 0.097 | -0.153 | 0.348 |  |
| **3D pronation** | **Initial position (°)** | Willems et al., 2007 | -0.058 | -0.309 | 0.192 |  |
|  | **Maximal position (°)** |  | 0.157 | -0.094 | 0.407 |  |
|  | **Pronation excursion (°)** |  | **0.488** | **0.234** | **0.740** | p<0.05 |
|  | **Push-off position (°)** |  | 0.152 | -0.099 | 0.403 |  |
|  | **Maxmimal pronation velocity (° s-1)** |  | 0.158 | -0.093 | 0.408 |  |
|  | **Mean pronation velocity (° s-1)** |  | -0.092 | -0.343 | 0.158 |  |
|  | **Timing of maximal pronation (% of stance phase)** |  | 0.054 | -0.197 | 0.304 |  |

Iliotibial band syndrome continuous plantar variables

| **Kinematic variable** | **Study ID** | **Effect size (ES)** |  | **Confidence interval** | | **Statistical significance reported in paper** |
| --- | --- | --- | --- | --- | --- | --- |
|  | |  |  | **Lower** | **Upper** |  |
| **Rearfoot motion** | **Rearfoot eversion peak** | Noehren et al., 2007 | -0.646 | -1.311 | 0.048 |  |
| **Rearfoot moment** | **Rearfoot inversion moment** | Noehren et al., 2007 | -0.210 | -0.880 | 0.471 |  |

Patellofemoral pain continuous plantar variables

| **Kinematic variable** | **Study ID** | **Effect size (ES)** |  | **Confidence interval** | | **Statistical significance reported in paper** |
| --- | --- | --- | --- | --- | --- | --- |
|  | |  |  | **Lower** | **Upper** |  |
| **Rearfoot motion** | **Rearfoot eversion angle** | Noehren et al., 2012 | -0.644 | -1.359 | 0.107 |  |

Patellofemoral pain discontinuous (nominal) kinematic variables

| **Plantar loading parameter** | **Study ID/Ref** | **Side** | **Experiment vs control** | **Risk ratio** | **95% CI** | |
| --- | --- | --- | --- | --- | --- | --- |
|  |  |  |  |  | **Lower** | **Upper** |
| **Maximum pronation angle (degree)** | **Hetsroni et al., 2006** | L | Q1 vs Q2 | 1.8889 | 0.8838 | 4.0368 |
|  |  |  | Q1 vs Q3 | 1.7173 | 0.8266 | 3.5678 |
|  |  |  | Q1 vs Q4 | 1.8889 | 0.8838 | 4.0368 |
|  |  |  | Q2 vs Q1 | 0.5294 | 0.2477 | 1.1314 |
|  |  |  | Q2 vs Q3 | 0.9092 | 0.3857 | 2.1434 |
|  |  |  | Q2 vs Q4 | 1.0000 | 0.4140 | 2.4154 |
|  |  |  | Q3 vs Q1 | 0.5823 | 0.2803 | 1.2097 |
|  |  |  | Q3 vs Q2 | 1.0999 | 0.4665 | 2.5930 |
|  |  |  | Q3 vs Q4 | 1.0999 | 0.4665 | 2.5930 |
|  |  |  | Q4 vs Q1 | 0.5294 | 0.2477 | 1.1314 |
|  |  |  | Q4 vs Q2 | 1.0000 | 0.4140 | 2.4154 |
|  |  |  | Q4 vs Q3 | 0.9092 | 0.3857 | 2.1434 |
|  |  | R | Q1 vs Q2 | 0.5000 | 0.1952 | 1.2805 |
|  |  |  | Q1 vs Q3 | 0.5455 | 0.2097 | 1.4185 |
|  |  |  | Q1 vs Q4 | 0.5000 | 0.1952 | 1.2805 |
|  |  |  | Q2 vs Q1 | 2.0000 | 0.7809 | 5.1221 |
|  |  |  | Q2 vs Q3 | 1.0909 | 0.5050 | 2.3568 |
|  |  |  | Q2 vs Q4 | 1.0000 | 0.4718 | 2.1195 |
|  |  |  | Q3 vs Q1 | 1.8333 | 0.7049 | 4.7679 |
|  |  |  | Q3 vs Q2 | 0.9167 | 0.4243 | 1.9804 |
|  |  |  | Q3 vs Q4 | 0.9167 | 0.4243 | 1.9804 |
|  |  |  | Q4 vs Q1 | 2.0000 | 0.7809 | 5.1221 |
|  |  |  | Q4 vs Q2 | 1.0000 | 0.4718 | 2.1195 |
|  |  |  | Q4 vs Q3 | 1.0909 | 0.5050 | 2.3568 |
| **Pronation range of motion (degree)** |  | L | Q1 vs Q2 | 1.3104 | 0.5949 | 2.8863 |
|  |  |  | Q1 vs Q3 | 1.2718 | 0.6049 | 2.6743 |
|  |  |  | Q1 vs Q4 | 1.1551 | 0.5506 | 2.4233 |
|  |  |  | Q2 vs Q1 | 0.7631 | 0.3465 | 1.6808 |
|  |  |  | Q2 vs Q3 | 0.9706 | 0.4209 | 2.2381 |
|  |  |  | Q2 vs Q4 | 0.8815 | 0.3830 | 2.0286 |
|  |  |  | Q3 vs Q1 | 0.7863 | 0.3739 | 1.6533 |
|  |  |  | Q3 vs Q2 | 1.0303 | 0.4468 | 2.3760 |
|  |  |  | Q3 vs Q4 | 0.9082 | 0.4123 | 2.0006 |
|  |  |  | Q4 vs Q1 | 0.8657 | 0.4127 | 1.8163 |
|  |  |  | Q4 vs Q2 | 1.1345 | 0.4929 | 2.6109 |
|  |  |  | Q4 vs Q3 | 1.1011 | 0.4999 | 2.4254 |
|  |  | R | Q1 vs Q2 | 0.8885 | 0.4177 | 1.8901 |
|  |  |  | Q1 vs Q3 | 1.0338 | 0.4615 | 2.3158 |
|  |  |  | Q1 vs Q4 | 1.6345 | 0.6581 | 4.0596 |
|  |  |  | Q2 vs Q1 | 1.1255 | 0.5291 | 2.3941 |
|  |  |  | Q2 vs Q3 | 1.1635 | 0.5368 | 2.5222 |
|  |  |  | Q2 vs Q4 | 1.8395 | 0.7624 | 4.4385 |
|  |  |  | Q3 vs Q1 | 0.9673 | 0.4318 | 2.1667 |
|  |  |  | Q3 vs Q2 | 0.8595 | 0.3965 | 1.8630 |
|  |  |  | Q3 vs Q4 | 1.5810 | 0.6266 | 3.9889 |
|  |  |  | Q4 vs Q1 | 0.6118 | 0.2463 | 1.5196 |
|  |  |  | Q4 vs Q2 | 0.5436 | 0.2253 | 1.3117 |
|  |  |  | Q4 vs Q3 | 0.6325 | 0.2507 | 1.5959 |

| **Time to maximum pronation (°)** |  | L | Q1 vs Q2 | 1.3104 | 0.5949 | 2.8863 |
| --- | --- | --- | --- | --- | --- | --- |
|  |  |  | Q1 vs Q3 | 1.2718 | 0.6049 | 2.6743 |
|  |  |  | Q1 vs Q4 | 1.1551 | 0.5506 | 2.4233 |
|  |  |  | Q2 vs Q1 | 0.7631 | 0.3465 | 1.6808 |
|  |  |  | Q2 vs Q3 | 0.9706 | 0.4209 | 2.2381 |
|  |  |  | Q2 vs Q4 | 0.8815 | 0.3830 | 2.0286 |
|  |  |  | Q3 vs Q1 | 0.7863 | 0.3739 | 1.6533 |
|  |  |  | Q3 vs Q2 | 1.0303 | 0.4468 | 2.3760 |
|  |  |  | Q3 vs Q4 | 0.9082 | 0.4123 | 2.0006 |
|  |  |  | Q4 vs Q1 | 0.8657 | 0.4127 | 1.8163 |
|  |  |  | Q4 vs Q2 | 1.1345 | 0.4929 | 2.6109 |
|  |  |  | Q4 vs Q3 | 1.1011 | 0.4999 | 2.4254 |
|  |  | R | Q1 vs Q2 | 0.8885 | 0.4177 | 1.8901 |
|  |  |  | Q1 vs Q3 | 1.0338 | 0.4615 | 2.3158 |
|  |  |  | Q1 vs Q4 | 1.6345 | 0.6581 | 4.0596 |
|  |  |  | Q2 vs Q1 | 1.1255 | 0.5291 | 2.3941 |
|  |  |  | Q2 vs Q3 | 1.1635 | 0.5368 | 2.5222 |
|  |  |  | Q2 vs Q4 | 1.8395 | 0.7624 | 4.4385 |
|  |  |  | Q3 vs Q1 | 0.9673 | 0.4318 | 2.1667 |
|  |  |  | Q3 vs Q2 | 0.8595 | 0.3965 | 1.8630 |
|  |  |  | Q3 vs Q4 | 1.5810 | 0.6266 | 3.9889 |
|  |  |  | Q4 vs Q1 | 0.6118 | 0.2463 | 1.5196 |
|  |  |  | Q4 vs Q2 | 0.5436 | 0.2253 | 1.3117 |
|  |  |  | Q4 vs Q3 | 0.6325 | 0.2507 | 1.5959 |
| **Pronation velocity (°/s)** |  | L | Q1 vs Q2 | 1.3000 | 0.5978 | 2.8273 |
|  |  |  | Q1 vs Q3 | 2.6265 | 0.9719 | 7.0983 |
|  |  |  | Q1 vs Q4 | 0.7647 | 0.3923 | 1.4906 |
|  |  |  | Q2 vs Q1 | 0.7692 | 0.3537 | 1.6729 |
|  |  |  | Q2 vs Q3 | 2.0204 | 0.7157 | 5.7038 |
|  |  |  | Q2 vs Q4 | 0.5882 | 0.2832 | 1.2216 |
|  |  |  | Q3 vs Q1 | 0.3807 | 0.1409 | 1.0289 |
|  |  |  | Q3 vs Q2 | 0.4949 | 0.1753 | 1.3973 |
|  |  |  | Q3 vs Q4 | **0.2911** | **0.1116** | **0.7593** |
|  |  |  | Q4 vs Q1 | 1.3077 | 0.6709 | 2.5490 |
|  |  |  | Q4 vs Q2 | 1.7000 | 0.8186 | 3.5306 |
|  |  |  | Q4 vs Q3 | **3.4347** | **1.3170** | **8.9577** |
|  |  | R | Q1 vs Q2 | **0.2694** | **0.0926** | **0.7835** |
|  |  |  | Q1 vs Q3 | **0.2525** | **0.0875** | **0.7290** |
|  |  |  | Q1 vs Q4 | 0.6734 | 0.1959 | 2.3145 |
|  |  |  | Q2 vs Q1 | **3.7125** | **1.2763** | **10.7988** |
|  |  |  | Q2 vs Q3 | 0.9375 | 0.4903 | 1.7926 |
|  |  |  | Q2 vs Q4 | **2.5000** | **1.0106** | **6.1844** |
|  |  |  | Q3 vs Q1 | **3.9600** | **1.3717** | **11.4324** |
|  |  |  | Q3 vs Q2 | 1.0667 | 0.5578 | 2.0396 |
|  |  |  | Q3 vs Q4 | **2.6667** | **1.0876** | **6.5384** |
|  |  |  | Q4 vs Q1 | 1.4850 | 0.4321 | 5.1039 |
|  |  |  | Q4 vs Q2 | **0.4000** | **0.1617** | **0.9895** |
|  |  |  | Q4 vs Q3 | **0.3750** | **0.1529** | **0.9195** |
